# DNA damage induced by topoisomerase inhibitors activates SAMHD1 and blocks HIV‐1 infection of macrophages

**DOI:** 10.15252/embj.201796880

**Published:** 2017-10-30

**Authors:** Petra Mlcochova, Sarah J Caswell, Ian A Taylor, Greg J Towers, Ravindra K Gupta

**Affiliations:** ^1^ Division of Infection and Immunity UCL London UK; ^2^ Macromolecular Structure Laboratory The Francis Crick Institute London UK; ^3^ Africa Health Research Institute Durban KwaZulu Natal South Africa

**Keywords:** DNA damage, HIV, integration, macrophage, SAMHD1, DNA Replication, Repair & Recombination, Immunology, Microbiology, Virology & Host Pathogen Interaction

## Abstract

We report that DNA damage induced by topoisomerase inhibitors, including etoposide (ETO), results in a potent block to HIV‐1 infection in human monocyte‐derived macrophages (MDM). SAMHD1 suppresses viral reverse transcription (RT) through depletion of cellular dNTPs but is naturally switched off by phosphorylation in a subpopulation of MDM found in a G1‐like state. We report that SAMHD1 was activated by dephosphorylation following ETO treatment, along with loss of expression of MCM2 and CDK1, and reduction in dNTP levels. Suppression of infection occurred after completion of viral DNA synthesis, at the step of 2LTR circle and provirus formation. The ETO‐induced block was completely rescued by depletion of SAMHD1 in MDM. Concordantly, infection by HIV‐2 and SIVsm encoding the SAMHD1 antagonist Vpx was insensitive to ETO treatment. The mechanism of DNA damage‐induced blockade of HIV‐1 infection involved activation of p53, p21, decrease in CDK1 expression, and SAMHD1 dephosphorylation. Therefore, topoisomerase inhibitors regulate SAMHD1 and HIV permissivity at a post‐RT step, revealing a mechanism by which the HIV‐1 reservoir may be limited by chemotherapeutic drugs.

## Introduction

Retroviruses must reverse transcribe their RNA into DNA and integrate nascent viral DNA into the host genome in order to replicate (Skalka & Katz, 2005; Lusic & Siliciano, 2017). Increasing evidence suggests that macrophage infection contributes to the reservoir of infected cells that persist and prevent cure of HIV/AIDS (Alexaki *et al*, 2008; Siliciano & Greene, 2011; Watters *et al*, 2013; Honeycutt *et al*, 2016). Integration may be recognized as a form of DNA damage, and the host DNA damage response (DDR) (Daniel *et al*, 1999; Jackson & Bartek, 2009) is critical for “gap repair” during the integration process. Indeed, there is increasing evidence that key DDR proteins are involved in retroviral infection, specifically during integration (Daniel *et al*, 2003; Ariumi *et al*, 2005; DeHart *et al*, 2005; Lau *et al*, 2005). Furthermore, proteins able to restrict HIV infection, including APOBEC3G and SAMHD1, have been linked to DNA damage responses (Leonard *et al*, 2013; Roberts *et al*, 2013; Clifford *et al*, 2014; Kretschmer *et al*, 2015).

SAMHD1 is a deoxynucleotide triphosphohydrolase (Goldstone *et al*, 2011), and the mechanism of HIV restriction is thought to be via depletion of dNTPs to levels that are insufficient for completion of retroviral reverse transcription (RT) (Goldstone *et al*, 2011; Lahouassa *et al*, 2012; Schmidt *et al*, 2015). The activity of SAMHD1 is thought to be regulated by CDK1/2‐mediated phosphorylation at amino acid T592 (Cribier *et al*, 2013; White *et al*, 2013; Antonucci *et al*, 2016; Mlcochova *et al*, 2017). Here, we link DNA damage in primary myeloid cells and cellular SAMHD1 activation, in the absence of a type I interferon response. We show that DNA damage induced by topoisomerase inhibitors activates p53 and p21, leading to SAMHD1 T592 dephosphorylation/activation. Activated SAMHD1 mediates a block to HIV infection, which occurs after the synthesis of full‐length HIV DNA. Importantly, the etoposide (ETO)‐induced inhibition of HIV‐1 can be fully abrogated by SAMHD1 depletion.

## Results

### DNA damage induces a post‐reverse transcription block to HIV‐1 in macrophages

It has been reported that HIV can induce DNA damage during integration into host DNA (Daniel *et al*, 2004). Moreover, certain reports suggest that integration can be enhanced by DNA damage induction (Ebina *et al*, 2012; Koyama *et al*, 2013). The cellular response to DNA damage also typically involves cell cycle arrest and activation of DNA damage repair (DDR) (Branzei & Foiani, 2008). We reported recently that HDAC inhibitors, which are known to induce cell cycle arrest and/or apoptosis, inhibit HIV‐1 infection in monocyte‐derived macrophages (MDM) through activation of SAMHD1 (Mlcochova *et al*, 2017).

**Figure 1:**
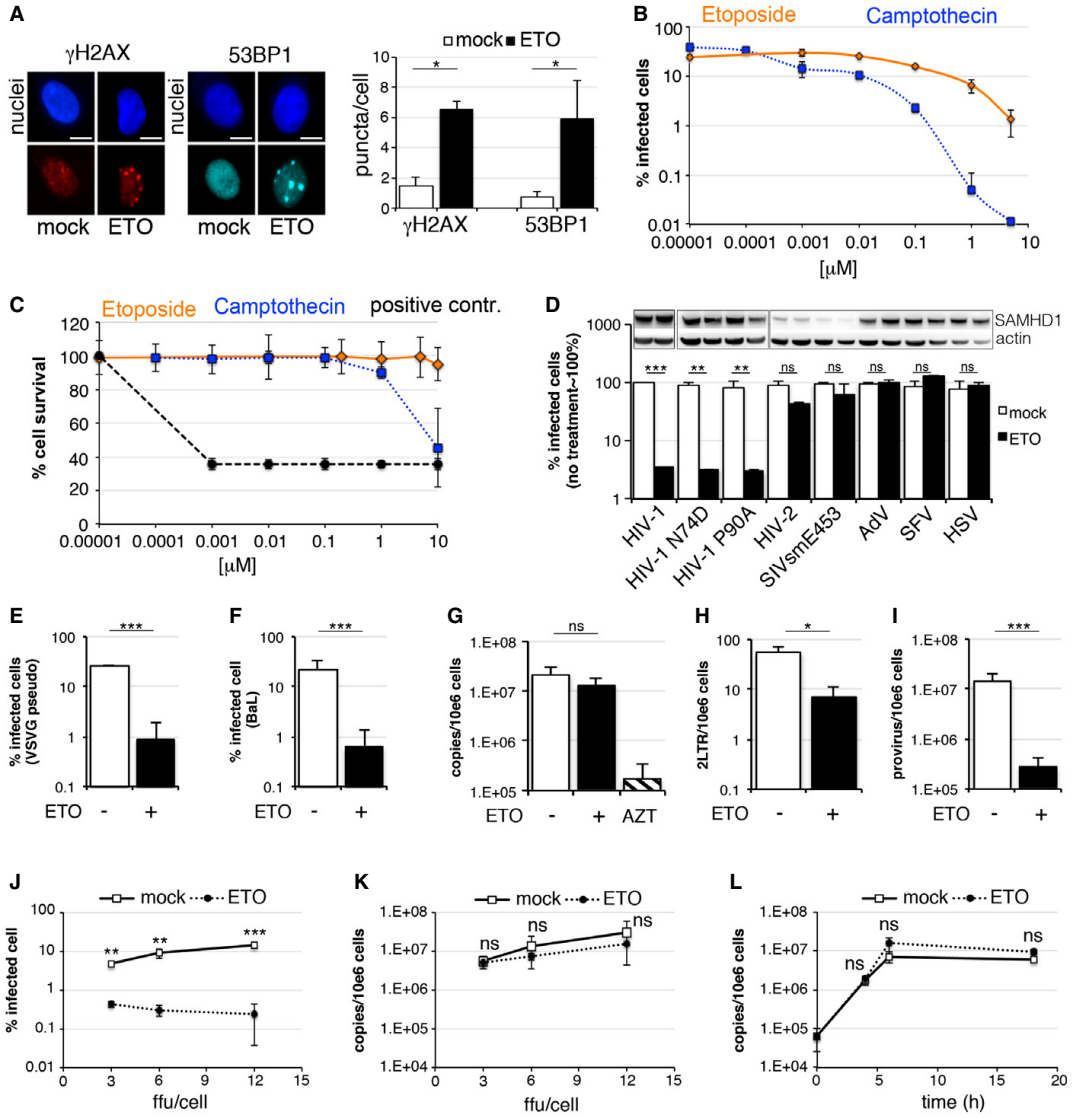
Etoposide/Camptothecin‐induced DNA damage inhibits HIV‐1 infection AMonocyte‐derived macrophages (MDM) were treated with 5 μM etoposide (ETO) for 18 h. Cells were stained for γH2AX and 53BP1 nuclear foci, acquired and analysed using the automated cell‐imaging system Hermes WiScan and ImageJ. On average, 10^4^ cells were acquired and analysed (*n* = 3, mean ± s.e.m.; *P‐value ≤ 0.05; paired *t*‐test). Scale bars, 10 μm.BMDM were treated with increasing concentrations of etoposide (ETO) and camptothecin (CTH) 18 h before infection. Cells were infected with VSV‐G pseudotyped HIV‐1 GFP in the presence of ETO/CTH, and 10^4^ cells in each experiment were recorded and analysed for GFP expression 48 h post‐infection using an automated cell‐imaging system Hermes WiScan and ImageJ (*n* = 3, mean ± s.e.m.).CMDM were treated with increasing concentrations of ETO and CTH for 66 h. Cells were stained to distinguish between live and dead cells using LIVE/DEAD fixable Dead cell stain protocol. Percentages of live/dead cells were determined using automated cell‐imaging system Hermes WiScan and ImageJ. Addition of 20% ethanol for 10 min into cells treated with ETO was used as positive control (*n* = 3, mean ± s.e.m.).DMDM were treated with 5 μM ETO for 18 h and infected in the presence of ETO with VSV‐G pseudotyped HIV‐1 GFP viruses wild‐type (wt) HIV‐1 capsid mutants (N74D or P90A), HIV‐2, SIV sooty mangabey (SIVsm E543), replication competent adenovirus type 5 AdV (AdV), Semliki Forest virus (SFV) and HSV‐1. The percentages of infected cells were determined using an automated cell‐imaging system Hermes WiScan and ImageJ and normalized to untreated control ˜100% (*n* = 3, mean ± s.e.m.; **P‐value ≤ 0.01; ***P‐value ≤ 0.001; (ns) non‐significant, paired *t*‐test). Cells from a representative donor were used for immunoblotting.EMDM were treated with 5 μM ETO for 18 h and infected in the presence of ETO with VSV‐G pseudotyped HIV‐1 GFP. 10^4^ cells were recorded and analysed for GFP expression 48 h post‐infection using an automated cell‐imaging system Hermes WiScan and ImageJ (*n* = 4, mean ± s.e.m.; ***P‐value ≤ 0.001, paired *t*‐test).FMDM were treated with 5 μM ETO for 18 h and infected in the presence of ETO with full‐length replication competent macrophage tropic HIV‐1 virus BaL. Cells were stained for intracellular p24, and the percentage of infected cells was quantified 48 h post‐infection by FACS (*n* = 3, mean ± s.e.m.; ***P‐value ≤ 0.001, paired *t*‐test).G–IMDM were treated with 5 μM ETO for 18 h and infected in the presence of ETO with HIV‐1 BaL and DNA isolated 18 h post‐infection for qPCR (*n* = 3, mean ± s.e.m.; *P‐value ≤ 0.05; ***P‐value ≤ 0.001; (ns) non‐significant, paired *t*‐test). (G) Late viral RT products. AZT: MDM treated with 20 μM AZT, a reverse‐transcriptase inhibitor, were used as control. (H) 2LTR circles. (I) Integrated copies of viral DNA, Alu‐Gag qPCR.JMDM were treated with 5 μM ETO for 18 h and infected with HIV‐1 BaL (3, 6, 12 ffu/cell). Cells were stained for intracellular p24, and the percentage of infected cells was quantified 48 h post‐infection by FACS (*n* = 3, mean ± s.e.m.; **P‐value ≤ 0.01; ***P‐value ≤ 0.001, paired *t*‐test).KMDM were treated with 5 μM ETO for 18 h, infected with HIV‐1 BaL (3, 6, 12 ffu/cell) and DNA isolated 18 h post‐infection for qPCR (*n* = 3, mean ± s.e.m.; (ns) non‐significant, paired *t*‐test).LMDM were treated with 5 μM ETO for 18 h, infected with HIV‐1 BaL and DNA isolated 4, 6 and 18 h post‐infection for qPCR (*n* = 3, mean ± s.e.m.; (ns) non‐significant, paired *t*‐test). Source data are available online for this figure.

Here, we investigate the impact of inducing DNA damage on HIV‐1 infection in MDM. We employed topoisomerase inhibitors, which block the unwinding of dsDNA leading to inhibition of fundamental biological processes including DNA replication, transcription, DNA repair and chromatin remodelling, by stabilizing the DNA‐single or ‐double breaks (Hsiang *et al*, 1989; Gorczyca *et al*, 1993). To test this, we treated MDM with 5 μM ETO for 18 h to confirm that ETO induced DNA damage, measured by staining for nuclear γH2AX or 53BP1 (Fig 1A) in uninfected cells. Further, we pretreated MDM with etoposide (ETO) or camptothecin (CTH), inhibitors of topoisomerase I and II, respectively. 18 h after treatment with titrations of inhibitors, we infected cells with VSV‐G pseudotyped HIV‐1 GFP and measured infection 48 h later by enumerating GFP‐positive cells. Both topoisomerase inhibitors blocked HIV‐1 infection in a dose‐dependent manner (Fig 1B). We then used the most effective, non‐cytotoxic, concentration of ETO (5 μM) (Fig 1C) to measure its effect on MDM infection by different viruses (Fig 1D). HIV‐1 wild‐type and capsid mutants N74D and P90A (known to use alternative host cofactors for nuclear translocation) were equally sensitive to topoisomerase inhibitors. However, HIV‐2 and SIVsm, which encode Vpx, and degrade SAMHD1, were insensitive to drug treatment. Infection by DNA viruses, adenovirus (AdV) and herpes simplex virus (HSV), and RNA virus Semliki Forest virus (SFV), was also insensitive to topoisomerase inhibition.

**Figure EV1:**
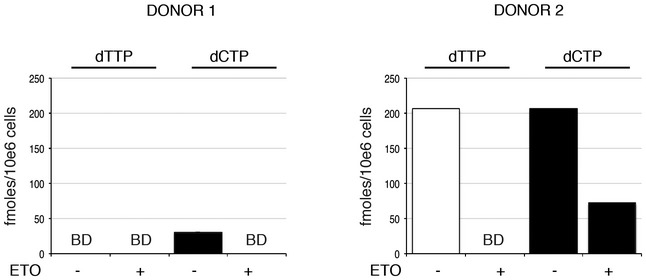
Quantification of dNTP levels in MDM treated with etoposide (ETO) Analysis of cellular dNTP levels in unstimulated and ETO‐treated MDM. dNTPs were extracted for quantification from two donors. The bar graph shows the amounts (fmoles per 10^6^ cells) of the indicated dNTP from each donor, with and without ETO treatment. BD: below level of detection.

We next investigated where in the HIV‐1 life cycle ETO acts. We treated MDM with 5 μM ETO for 18 h and infected cells with VSV‐G pseudotyped HIV‐1 GFP (known to use an endocytic entry route) or wild‐type macrophage and CCR5 tropic HIV‐1 isolate BaL and determined infection (Fig 1E and F). Both viruses were equally sensitive to the ETO‐mediated inhibition of viral infection, suggesting independence of drug sensitivity from viral route of entry. MDM infected with HIV‐1 BaL were used for DNA isolation and qPCR to determine efficiency of reverse transcription (Fig 1G), viral 2LTR circle formation (a measure of nuclear entry) (Fig 1H; De Iaco *et al*, 2013) and viral integration by Alu‐gag PCR (Fig 1I). Surprisingly, reverse transcription was not affected by ETO treatment (Fig 1G), but we observed a decrease in 2LTR circles (Fig 1H) and viral integration (Fig 1I). We also confirmed that ETO did not affect RT products at increasing MOI (determined as ffu/target cell), even though infection was significantly decreased (Fig 1J and K). Importantly, ETO did not have any effect on RT products over a time‐course (Fig 1L). In addition, although dNTP levels are already very low in MDM, we detected a decrease in dTTP and dCTP after ETO treatment (Fig EV1).

### DNA damage‐induced HIV‐1 block is SAMHD1 dependent in macrophages

**Figure 2:**
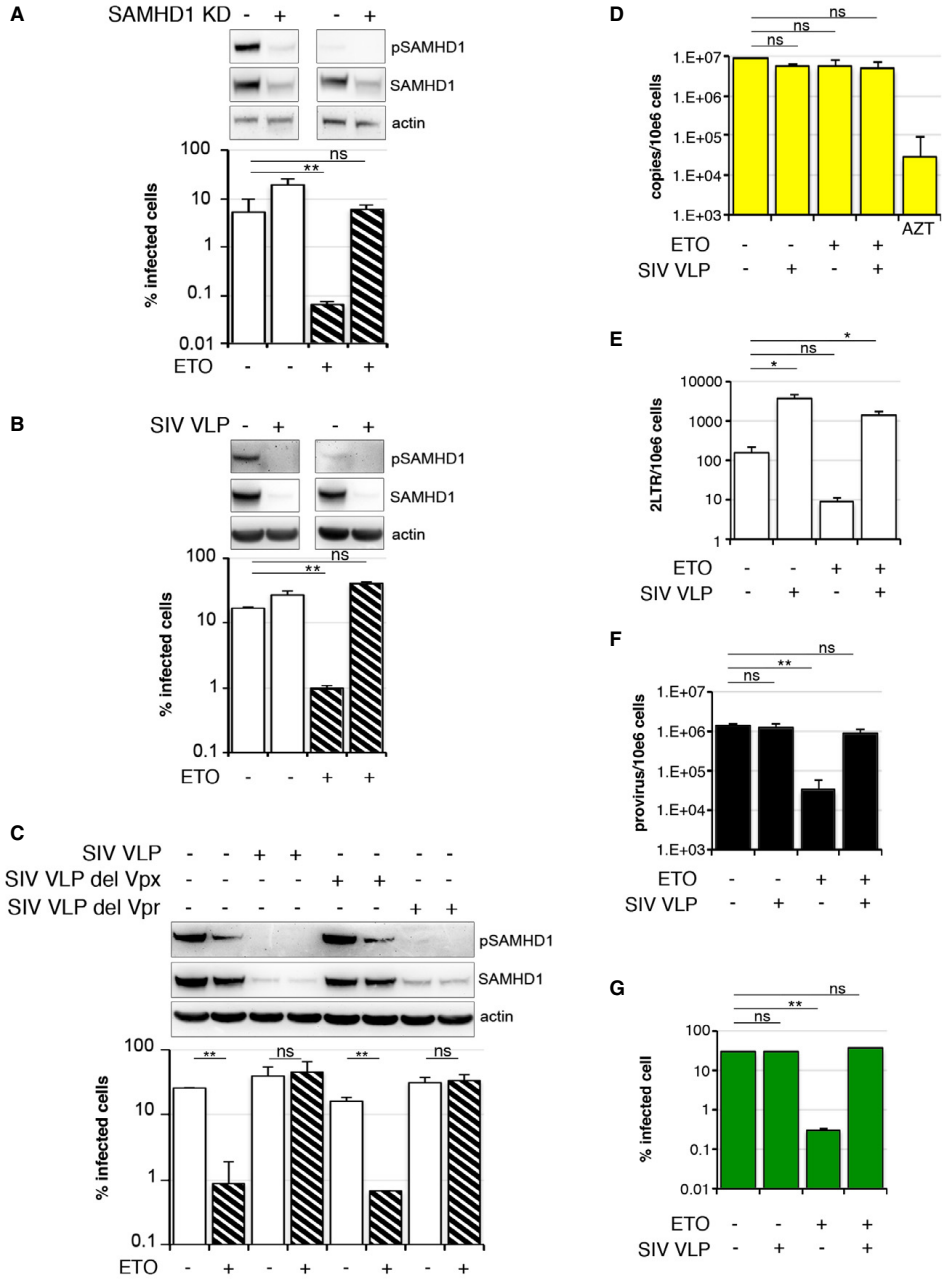
SAMHD1 inhibits HIV‐1 at a post‐RT step following DNA damage AMDM were transfected with control or pool of SAMHD1 (KD) siRNA and 3 days later treated with 5 μM ETO and infected in the presence of ETO with VSV‐G pseudotyped HIV‐1 GFP 18 h later. Cells from a representative donor were used for immunoblotting. The percentage of infected cells was quantified by the automated cell‐imaging system Hermes WiScan and ImageJ 48 h post‐infection (*n* = 3, mean ± s.e.m.; **P‐value ≤ 0.01; (ns) non‐significant, paired *t*‐test).BMDM were treated with 5 μM ETO for 18 h and co‐infected in the presence of ETO with VSV‐G HIV‐1 GFP and SIVmac virus‐like particles containing Vpx/Vpr (SIV VLP). Cells from a representative donor were used for immunoblotting. The percentage of infected cells was quantified by the Hermes WiScan and ImageJ 48 h post‐infection (*n* = 3, mean ± s.e.m.; **P‐value ≤ 0.01; (ns) non‐significant, paired *t*‐test).CMDM were treated with 5 μM ETO for 18 h and co‐infected in the presence of ETO with VSV‐G HIV‐1 GFP and SIVmac virus‐like particles containing Vpx/Vpr (SIV VLP) or deleted for Vpx (SIV VLP del Vpx) or deleted for Vpr (SIV VLP del Vpr). Cells from a representative donor were used for immunoblotting. The percentage of infected cells was quantified by the automated cell‐imaging system Hermes WiScan and ImageJ 48 h post‐infection (*n* = 4, mean ± s.e.m.; **P‐value ≤ 0.01; (ns) non‐significant, paired *t*‐test).D–FMDM were treated with 5 μM ETO for 18 h and co‐infected in the presence of ETO with HIV‐1 BaL and SIVmac virus‐like particles containing Vpx/Vpr (SIV VLP). DNA was isolated 18 h post‐infection for qPCR quantification of (F) late RT products; (G) 2LTR circles; (H) integrated viral DNA (*n* = 3, mean ± s.e.m.; *P‐value ≤ 0.05; **P‐value ≤ 0.01; (ns) non‐significant, paired *t*‐test). (D) Late viral RT products. AZT: MDM treated with 20 μM AZT, a reverse‐transcriptase inhibitor, were used as control. (E) 2LTR circles. (F) Integrated copies of viral DNA, Alu‐Gag qPCR.GThe percentage of infected cells was quantified by Hermes WiScan and ImageJ 48 h post‐infection (*n* = 3, mean ± s.e.m.; **P‐value ≤ 0.01; (ns) non‐significant, paired *t*‐test). Source data are available online for this figure.

**Figure EV2:**
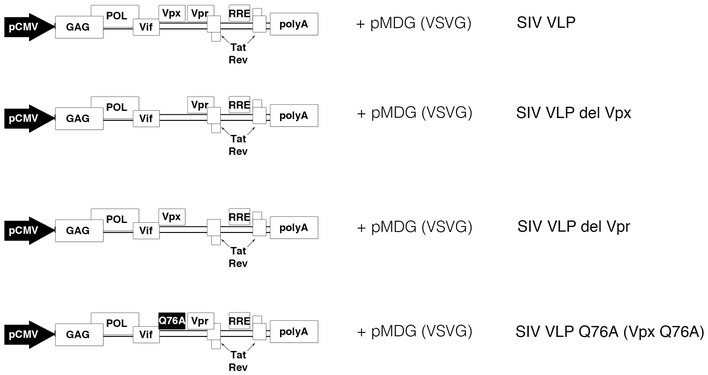
Schematic representation of SIVmac VLP used in the study Schematic representation of the packaging plasmids derived from SIVmac251. SIVmac virus‐like (SIV VLP) particles were prepared as described in Materials and Methods.

**Figure EV3:**
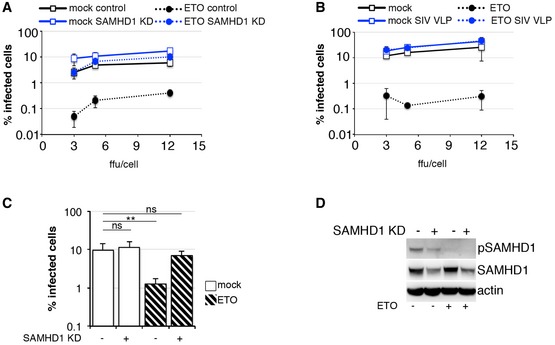
SAMHD1 inhibits HIV‐1 following DNA damage MDM were transfected with control or pool of SAMHD1 (KD) siRNA and 3 days later treated with 5 μM ETO and infected in the presence of ETO with HIV‐1 BaL (3, 5, 12 ffu/cell). Cells were stained for intracellular p24, and the percentage of infected cells was quantified 48 h post‐infection by FACS (*n* = 3, mean ± s.e.m.).MDM were treated with 5 μM ETO for 18 h and co‐infected in the presence of ETO with HIV‐1 BaL (3, 5, 12 ffu/cell) and SIVmac virus‐like particles containing Vpx/Vpr (SIV VLP). Cells were stained for intracellular p24, and the percentage of infected cells was quantified 48 h post‐infection by FACS (*n* = 3, mean ± s.e.m.).MDM were transfected with control or pool of SAMHD1 (KD) siRNA and 3 days later treated with 5 μM ETO for 18 h and infected in the presence of ETO with VSV‐G HIV‐1 GFP. The percentage of infected cells was quantified by the automated cell‐imaging system Hermes WiScan and ImageJ 48 h post‐infection (*n* = 3, mean ± s.e.m.; **P‐value ≤ 0.01; (ns) non‐significant, paired *t*‐test).Cells from a representative donor were used for immunoblotting.

We observed that lentiviruses encoding Vpx (HIV‐2 and SIVsm), a SAMHD1 antagonist (Hrecka *et al*, 2011; Laguette *et al*, 2011), were insensitive to ETO in MDM suggesting that SAMHD1 might be responsible for the effect of ETO/CTH on HIV‐1 infection (Fig 1D). To test this, we treated MDM with 5 μM ETO, infected cells with HIV‐1 and assayed phosphorylation of SAMHD1 at T592 by immunoblot (Fig 2A–C). We found that SAMHD1 is phosphorylated in untreated MDM allowing efficient HIV infection, confirming previous data (Mlcochova *et al*, 2017). Addition of ETO led to loss of SAMHD1 phosphorylation and reduced HIV‐1 infectivity. As expected, the inhibitory effect of ETO on HIV infection was lost after siRNA‐mediated SAMHD1 depletion (SAMHD1 KD) (Fig 2A) or treatment of MDM with SIVmac virus‐like particles containing Vpx/Vpr (SIV VLP; Figs 2B and EV2), which was confirmed by a dose titration of HIV‐1 virus on SAMHD1 KD (Fig EV3A) and SIV VLP‐treated cells (Fig EV3B) and by infecting control and SAMHD1 KD cells at MOI achieving equal percentage of infected cells (Fig EV3C and D). Importantly, the rescue was independent of the SIV Vpr protein, as evidenced by wild‐type activity of SIV VLP deleted for Vpr (Figs 2C and EV2) but completely dependent on presence of Vpx. To investigate which step of the virus life cycle was inhibited by ETO, and consequently rescued by SAMHD1 depletion, we infected MDM with HIV‐1 BaL with and without co‐infection with SIV VLP and isolated DNA 18 h post‐infection to measure late RT products (Fig 2D), 2LTR circles (Fig 2E) and integration products (Fig 2F) under conditions of FCS culture where SAMHD1 is phosphorylated in the absence of ETO. Infection was measured in parallel samples 48 h post‐viral challenge (Fig 2G). Critically, the post‐RT block seen on ETO treatment was abrogated by SAMHD1 depletion. We conclude that HIV inhibition following ETO treatment is mediated through SAMHD1 and impacts both nuclear import and viral integration, but not late viral DNA synthesis.

Surprisingly, we noted that HIV‐1 2LTR circles were increased by nearly 20‐fold by co‐infection with SIV VLP (Fig 2E) in the absence of ETO. However, this increase in 2LTR circles was not mirrored by an increase in integrated proviral DNA (Fig 2E and F).

### SIV bearing Vpx mutant Q76A rescues HIV‐1 infection following DNA damage

**Figure 3:**
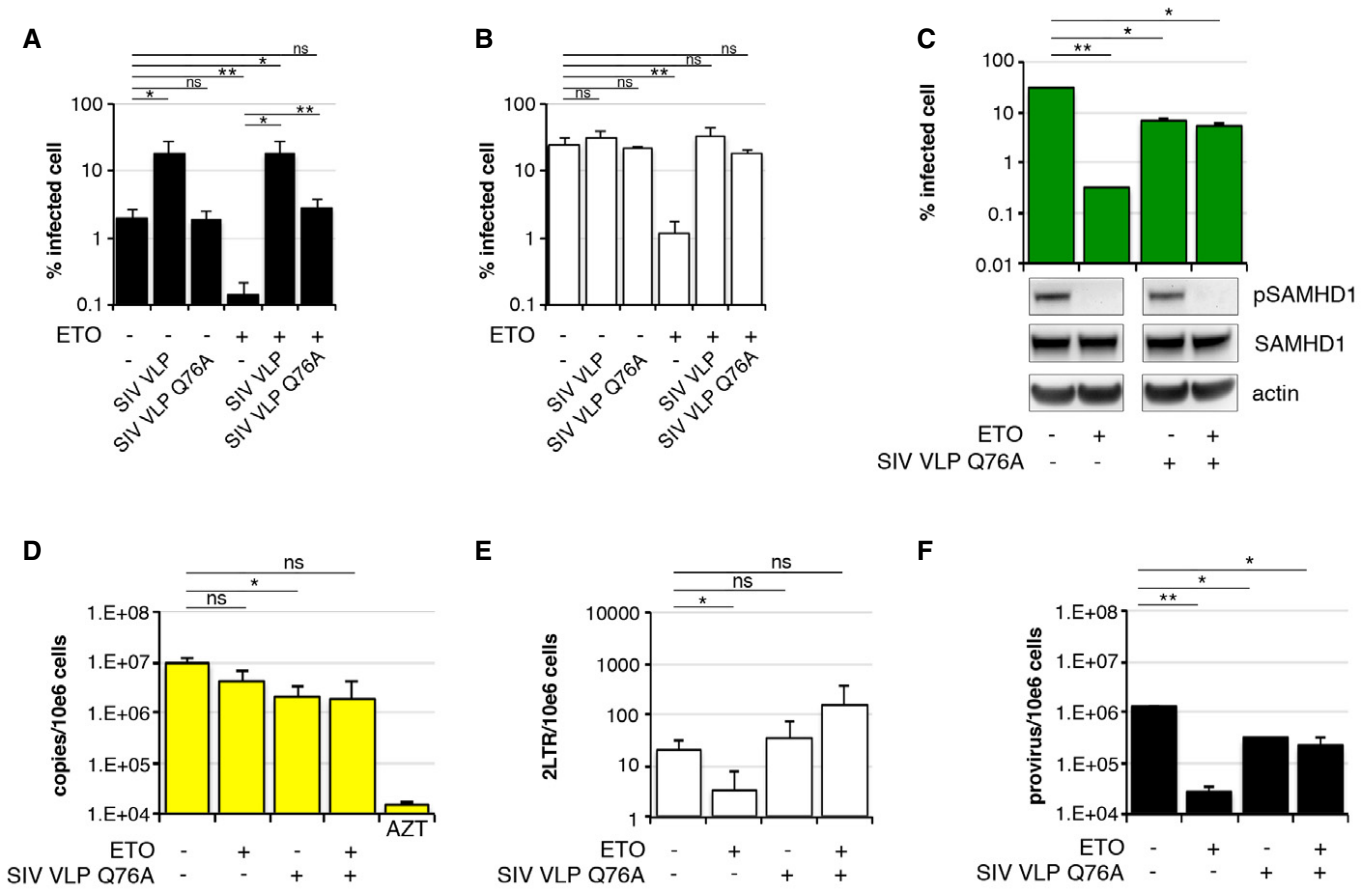
Vpx Q76A rescues DNA damage‐induced block to HIV‐1 infection A, BMDM were treated with 5 μM ETO for 18 h and co‐infected in the presence of ETO with VSV‐G HIV‐1 GFP and SIVmac virus‐like particles containing Vpx wild‐type (WT)/Vpr or Vpx Q76A mutant/Vpr (SIV VLP Q76A) (*n* = 3, mean ± s.e.m.; *P‐value ≤ 0.05; **P‐value ≤ 0.01; (ns) non‐significant, paired *t*‐test). (A) MDM were differentiated and cultured in human serum instead of FCS. (B) MDM were differentiated and cultured in FCS. A standard culture condition used in all experiments. See Materials and Methods.C–FMDM were treated with 5 μM ETO for 18 h and co‐infected in the presence of ETO with HIV‐1 BaL and SIV VLP Q76A. DNA was isolated 18 h post‐infection for qPCR quantification of (D) late RT products; (E) 2LTR circles; (F) integrated viral DNA (*n* = 3, mean ± s.e.m.; *P‐value ≤ 0.05; **P‐value ≤ 0.01; (ns) non‐significant, paired *t*‐test). (C) The percentage of infected cells was quantified by the automated cell‐imaging system Hermes WiScan and ImageJ 48 h post‐infection. Cells from a representative donor were used for immunoblotting. (D) Late viral RT products. AZT: MDM treated with 20 μM AZT, a reverse‐transcriptase inhibitor, were used as control. (E) 2LTR circles. (F) Integrated copies of viral DNA, Alu‐Gag qPCR. Source data are available online for this figure.

**Figure EV4:**
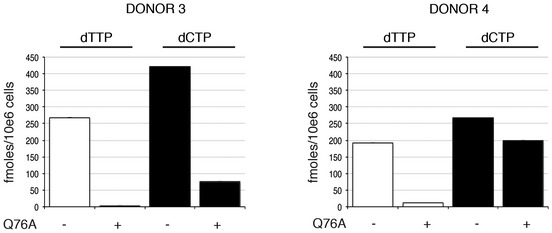
Quantification of dNTP levels in MDM following treatment with SIV VLP Q76A Analysis of cellular dNTP levels in MDM and MDM expressing SIV Vpx(Q76A). dNTPs were extracted for quantification from two donors. The bar graph shows the amounts (fmoles per 10^6^ cells) of the indicated dNTP from the MDM of each donor, before and after the introduction of SIV Vpx(Q76A) in SIV VLPs.

To further probe the mechanism of ETO on HIV‐1 infection, we treated MDM with 5 μM ETO for 18 h, co‐infected cells with HIV‐1 and SIV VLP (bearing Vpx WT/Vpr WT) or SIV VLP Q76A (bearing Vpx Q76A mutant/Vpr WT) and measured infection 48 h later. The Vpx mutant Q76A maintains the ability to interact with SAMHD1, but in previous studies did not rescue HIV‐1 infection from SAMHD1, possibly because it cannot recruit DCAF1 to degrade SAMHD1 (Srivastava *et al*, 2008; Hrecka *et al*, 2011; Laguette *et al*, 2011; Reinhard *et al*, 2014). We used MDM cultured in human serum (low dNTP levels, dephosphorylated SAMHD1) and in FCS (high dNTP levels, phosphorylated SAMHD1) (Mlcochova *et al*, 2017) and co‐infected cells with HIV‐1 and SIV VLP or SIV VLP Q76A (Fig 3A and B). SIV VLP but not SIV VLP Q76A increased infection in MDM (Fig 3A and B). By contrast, ETO‐mediated inhibition of HIV‐1 infection had been rescued by both SIV VLP and SIV VLP Q76A (Fig 3A and B). ETO caused dephosphorylation of SAMHD1 and blocked HIV‐1 infection in MDM. However, this block to infection was abrogated by SIV VLP Q76A, even though SAMHD1 was not degraded. As expected, dNTP levels following treatment with SIV VLP Q76A infected cells were not increased (Fig EV4). Critically, SAMHD1 was dephosphorylated (active) in the presence of ETO and SIV VLP Q76A (Fig 3C) suggesting inhibition of the active form of SAMHD1 by the Vpx mutant without manipulation of activation by T592 phosphorylation or degradation.

To confirm which step of the virus life cycle was inhibited by ETO and rescued by SIV VLP Q76A, we infected MDM with HIV‐1 BaL with and without co‐infection with SIV VLP Q76A and measured infection (Fig 3C), late RT products (Fig 3D), 2LTR circles (Fig 3E) and integration products (Fig 3F). We detected a ~3‐ to 5‐fold decrease in RT, integration of viral products and infection in the presence of Vpx Q76A. This was independent of ETO treatment (Fig 3C–F). Importantly, there was no reduction in reverse transcription but substantial restriction of integrated provirus formation after ETO treatment, which was rescued by co‐infection with SIV VLP Q76A (Fig 3A–F). Interestingly, we noted that HIV‐1 2LTR circles were not increased by co‐infection with SIV VLP Q76A (Fig 3E) in the absence of ETO. These data suggest that following DNA damage, SAMHD1 can be antagonized without being degraded or phosphorylated at T592.

### ETO‐induced DNA damage does not trigger type I IFN responses in MDM

Recent evidence suggests that DNA damage activates the type I interferon system to anti‐microbial responses (Brzostek‐Racine *et al*, 2011; Hartlova *et al*, 2015). As SAMHD1 was shown to mediate spontaneous expression and release of IFN when mutated (Crow & Manel, 2015) or deleted (Behrendt *et al*, 2013), we investigated the possibility that DNA damage induction could activate a type I IFN response and block HIV‐1 infection in MDM.

**Figure 4:**
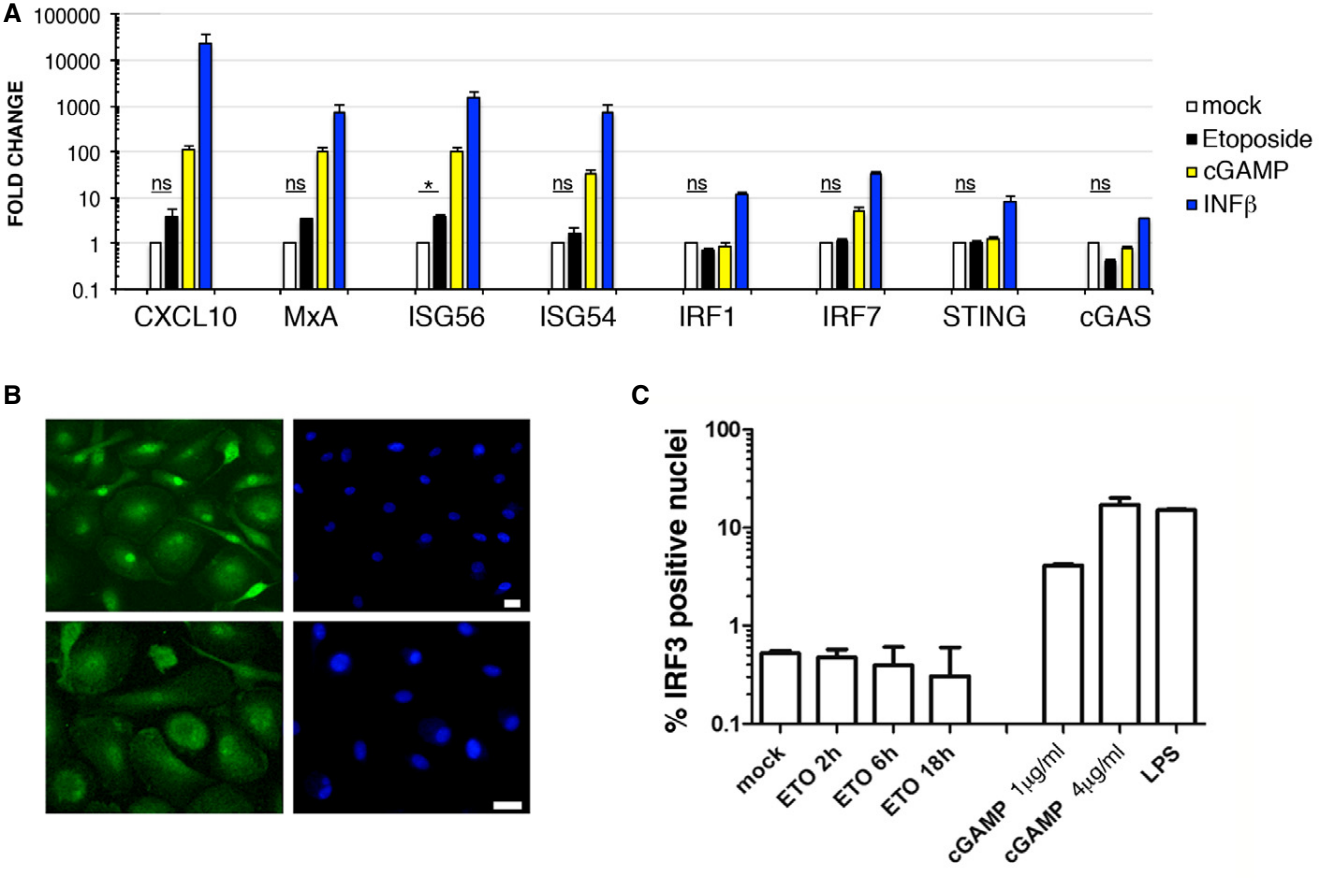
ETO‐induced DNA damage does not activate type I IFN response in MDM MDM were treated with 5 μM ETO for 18 h, 3 μg/ml cGAMP and 10 ng/ml IFN‐β for 18 h. RNA was isolated and qPCR performed for selected genes using TaqMan assays. Expression levels of target genes were normalized to GAPDH (*n* = 3, mean ± s.e.m.; *P‐value ≤ 0.05; (ns) non‐significant, paired *t*‐test).MDM were treated with 5 μM ETO or cGAMP for 18 h or 100 ng/ml LPS for 2 h. Cells were stained and analysed for IRF3 translocation (green) into the nucleus (blue). Scale bars: 10 μm.Quantification of nuclei positive for IRF3 staining (*n* = 3, mean ± s.e.m.).

To test this, we treated MDM with 5 μM ETO for 18 h to induce DNA damage. As a positive control for innate immune triggering, we treated MDM with cGAMP, the product of the activated DNA sensor cGAS (Sun *et al*, 2013; Wu *et al*, 2013) or IFN‐β for 18 h. We then isolated RNA from the cells and tested for specific interferon‐stimulated gene (ISG) transcripts (Fig 4A). None of the ISG transcripts tested was strongly elevated after ETO exposure of MDM. Moreover, an immunofluorescence/translocation assay detected 5–20% of IRF3‐positive nuclei in MDM after treatment with cGAMP and LPS (positive controls), consistent with activation of innate immune responses. However, there was no IRF3 translocation to the nucleus after 2, 6 or 18 h of ETO treatment (Fig 4B and C), suggesting that a type I IFN response is not strongly activated after ETO‐induced DNA damage in MDM.

### DNA damage promotes a G0 state and regulates SAMHD1 phosphorylation via p53 and p21

**Figure 5:**
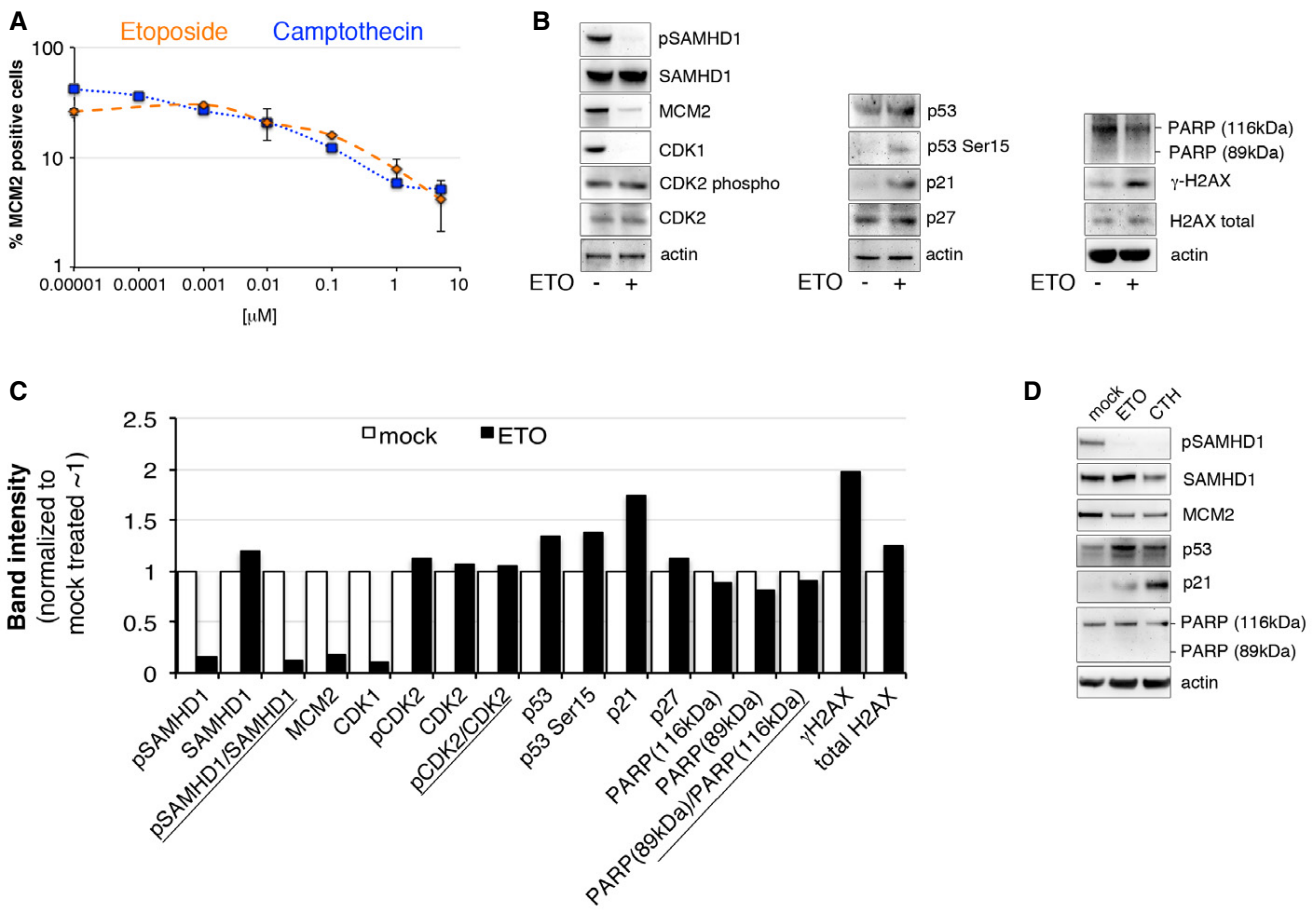
ETO regulates SAMHD1 phosphorylation through the p53, p21 pathway MDM were treated with increasing concentrations of ETO and CTH. Cells were stained for MCM2 expression, acquired and analysed using the automated cell‐imaging system Hermes WiScan and ImageJ. On average, 10^4^ cells were acquired (*n* = 3, mean ± s.e.m.).MDM were treated with 5 μM ETO, lysed and immunoblotting performed to detect cell cycle/cell cycle arrest and DNA damage‐associated proteins.Quantification of specific proteins band intensities from immunoblot in panel (B) using a CCD camera. Intensities of protein bands were normalized to intensity of actin protein band.MDM were treated with 5 μM ETO or 0.01 μM CTH for 18 h, lysed and immunoblotting performed to detect cell cycle/cell cycle arrest and DNA damage‐associated proteins.Data information: (B–D) Each panel shows a representative example of three independent experiments.Source data are available online for this figure.

**Figure 6:**
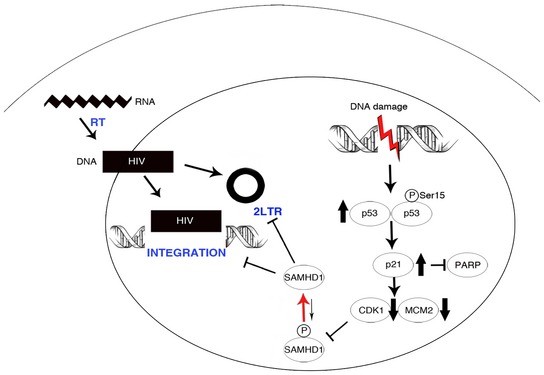
Proposed mechanism of action for ETO/CTH and SAMHD1 regulation ETO/CTH‐induced DNA damage appears to promote a p53/p21‐dependent pathway. This is associated with cell cycle arrest, evidenced by decrease in MCM2 expression. Furthermore, CDK1—responsible for SAMHD1 phosphorylation—is downregulated. As a result, SAMHD1 is not phosphorylated and becomes active against HIV‐1 infection. Dephosphorylated, active SAMHD1 induced by DNA damage appears to block 2LTR and provirus formation.

In the absence of general type I IFN responses after ETO treatment, we hypothesized that the restriction of HIV‐1 is mediated entirely by dephosphorylation and activation of SAMHD1. Recent evidence demonstrated that cell cycle status regulates phosphorylation of SAMHD1 (Cribier *et al*, 2013; Badia *et al*, 2016; Mlcochova *et al*, 2017). G0 state macrophages encode active, dephosphorylated SAMHD1, but SAMHD1 is phosphorylated and deactivated by CDK1 in a subpopulation of macrophages in a G1‐like state (Mlcochova *et al*, 2017). To test whether ETO altered cell cycle status and therefore activated SAMHD1 through the same pathway, we treated MDM with ETO and CTH and measured the proportion of cells in G0 or G1 by detection of MCM2, a marker expressed throughout the cell cycle but not in G0. In fact, the proportion of MDM expressing MCM2 and thus being in a G1‐like state was significantly reduced after ETO/CTH treatment, indicative of cells returning to G0, a state non‐permissive to HIV‐1 infection (Fig 5A). We mapped the pathway leading to SAMHD1 dephosphorylation and HIV restriction using immunoblotting (Fig 5B–D). ETO induced DNA damage, as measured by an increase in γH2AX, and resulted in increased expression of p53 and p53 phosphorylation at Ser15 (Fig 5B–D). We also observed increased expression of p21 but not p27 protein. Moreover, absence of PARP cleavage (Fig 5B–D) suggested lack of apoptosis, in addition supported by the lack of cell death measured using cell viability/cell survival analysis (Fig 1C). Loss of HIV‐1 permissivity following ETO treatment also correlated with loss of CDK1 and SAMHD1 activation by dephosphorylation. No increase in expression or phosphorylation of CDK2 was detected (Fig 5B and C). The same results were obtained with the topoisomerase I inhibitor CTH (Fig 5D). These data suggest that a DNA damage‐induced block to HIV infection in human macrophages is mediated through cell cycle arrest activated by a p53/p21/CDK1 pathway culminating in activation of SAMHD1 by dephosphorylation (Fig 6).

## Discussion

DNA damage and repair have been implicated in regulation of HIV‐1 infection, although such reports have opposed each other, likely due to the different cellular backgrounds used (Daniel *et al*, 2003; Ariumi *et al*, 2005; DeHart *et al*, 2005; Lau *et al*, 2005). Indeed, the effect of DNA repair kinases including ATM, ATR, DNA‐PK on retroviral infection appears to be variable from cell line to cell line (Yang *et al*, 2010). Here, we present evidence that DNA damage induced by topoisomerase inhibitors used in cancer treatment is associated with SAMHD1 dependent inhibition of HIV‐1 infection in primary human MDM. ETO induced activation of SAMHD1 by dephosphorylation and activated its ability to restrict HIV‐1. By contrast, it did not restrict HIV‐2 and SIVsm infections because they encode the SAMHD1 antagonist protein Vpx. SAMHD1 was also previously shown to inhibit HSV‐1 infection by limiting viral DNA replication, but this inhibition was reported to be independent from SAMHD1 T592 phosphorylation status (Kim *et al*, 2013). Consistent with this observation, we could not detect any effect of ETO on HSV infection in MDM (Fig 1C).

Considerable evidence indicates that SAMHD1 is a dNTP triphosphohydrolase limiting levels of dNTPs and thus retroviral reverse transcription (Goldstone *et al*, 2011; Lahouassa *et al*, 2012; Schmidt *et al*, 2015; Antonucci *et al*, 2016) or herpes virus DNA replication (Kim *et al*, 2013). Nevertheless, other reports have suggested that there might be a second mechanism of SAMHD1 restriction independent of dNTP regulation (White *et al*, 2013; Bhattacharya *et al*, 2016; Welbourn, 2016). Intriguingly, ETO‐induced DNA damage was associated with a decrease in dNTP levels, but not with decrease in products of reverse transcription in MDM as might be expected if this mode of SAMHD1‐mediated restriction is the result of regulation of overall cellular dNTP levels and limitation of reverse transcription. Given RT takes place in the cytoplasm, it might be possible that adequate dNTP levels for RT are maintained in the cytoplasm following ETO treatment. Instead, we observed reduced 2LTR circle formation and integrated proviral DNA, indicating a nuclear translocation or integration defect following ETO treatment.

It has been reported that low dNTP levels found in macrophages can restrict DNA gap repair during HIV‐1 integration (Van Cor‐Hosmer *et al*, 2013) and therefore SAMHD1 could play role in integration through its dNTP triphosphohydrolase activity during this process. Both, formation of 2LTR circles and the gap repair during HIV‐1 integration into host genome are recognized by cells as DNA damage and are repaired by host cell DNA damage repair (DDR) machinery (Li *et al*, 2001; Skalka & Katz, 2005). DDR is highly localized into DNA damage nuclear foci (Rothkamm *et al*, 2015) and it might be possible that SAMHD1 alters dNTP concentration locally at DNA damage foci where gap repair takes place. Importantly, SAMHD1 was reported to localize to DNA damage foci in HeLa cells (Clifford *et al*, 2014), and therefore SAMHD1 could play a direct role locally at the site of HIV integration.

The Vpx Q76A variant that is able to interact with SAMHD1, but not DCAF1, fails to cause SAMHD1 degradation (Srivastava *et al*, 2008; Hrecka *et al*, 2011) and fails to increase dNTP levels in dendritic cells (Reinhard *et al*, 2014). Concordantly, we show that Vpx Q76A also fails to increase dNTP levels in MDM. However, we find that this mutant Vpx Q76A is still able to rescue HIV‐1 from the effect of ETO even though SAMHD1 protein persists in its active dephosphorylated form. Depletion of SAMHD1 (Fig 2A) illustrates that the effect of ETO on HIV‐1 infection in MDM is SAMHD1 dependent. We suggest that Vpx Q76A interaction with SAMHD1 is sufficient to disturb a local SAMHD1 restriction function acting post‐RT.

Our experiments with ETO suggest that modulation of dNTP levels can be independent from SAMHD1 restriction, supported by our observation that Vpx Q76A cannot increase dNTP levels but still rescues HIV‐1 infection in the presence of ETO. These data are consistent with reports of dNTP independent restriction by SAMHD1 (Bhattacharya *et al*, 2016; Welbourn, 2016). We speculate that SAMHD1 may bind viral DNA directly or indirectly and therefore mediate post‐RT restriction. Further investigation is warranted to uncover the mechanism at play.

Intriguingly, co‐infection of MDM with HIV and SIV VLP, but not with SIV VLP Q76A, led to an increase in 2LTR circles in untreated cells, reminiscent of observations made in monocyte‐derived dendritic cells (Reinhard *et al*, 2014). This observation remains as yet unexplained but suggests that Vpx can distinguish processes leading to 2LTR circle from provirus formation in macrophages.

Recent evidence suggest that DNA damage activates the interferon type I system for anti‐microbial responses in murine BMDM (Brzostek‐Racine *et al*, 2011; Hartlova *et al*, 2015) or in the human macrophage‐like THP‐1 cell line (Brzostek‐Racine *et al*, 2011). Given that SAMHD1 mutation (Crow & Manel, 2015) or deletion (Behrendt *et al*, 2013) has been associated with spontaneous expression and release of IFN *in vivo* we investigated type I IFN responses in human monocyte‐derived MDM after ETO‐induced DNA damage. We were unable to detect any large changes to expression of a variety of interferon‐stimulated genes. We conclude that type I IFN responses are not significantly activated after ETO‐induced DNA damage in MDM and are therefore not responsible for the block to infection seen.

In the absence of type I IFN related changes we were able to demonstrate that the ETO‐induced block is mediated by SAMHD1 T592 phosphorylation. Activation of p53 and the downstream p21 lead to decreased expression of CDK1, the key kinase in SAMHD1 phosphorylation (Cribier *et al*, 2013; Mlcochova *et al*, 2017). As expected from our previous data this loss of CDK1 activity and SAMHD1 phosphorylation was associated with transition of G1‐like MDM back into G0 state without induction of apoptosis. In this state, SAMHD1 was dephosphorylated and able to restrict HIV‐1.

In summary, we propose a mechanism where ETO‐induced DNA damage induces SAMHD1 dephosphorylation via a canonical p53 and p21 pathway in macrophages. As consequence of this regulation, activated dephosphorylated SAMHD1 mediates a block to HIV‐1 nuclear import and integration in MDM. ETO and related chemotherapeutic agents are used in HIV infected individuals for malignancies such as lymphoma (Little *et al*, 2003); future work could include exploration of the effect of subsequent DNA damage responses on protection of myeloid target cells from HIV infection. Such insights might assist in design of novel therapeutic interventions, particularly for persistent central nervous system reservoirs that primarily involve macrophages or related myeloid lineage cells.

## Materials and Methods

### Reagents, inhibitors, antibodies and plasmids

Tissue culture media and supplements were obtained from Invitrogen (Paisley, UK), and tissue culture plastic was purchased from TPP (Trasadingen, Switzerland). FCS (FBS) was purchased from Biosera (Boussens, France) and Sigma (Sigma, St. Louis, USA). Human serum from human male AB plasma was of USA origin and sterile‐filtered (Sigma). All chemicals, etoposide and campthotecin were purchased from Sigma (St. Louis, MO, USA) unless indicated otherwise. Antibodies used were anti‐cdc2 (Cell Signaling Technology, Beverly, USA); anti‐CDK2 (H‐298, Santa Cruz Biotechnology); anti‐pCDK2(Thr160) (Bioss Inc., MA, USA); anti‐SAMHD1 (ab67820, Abcam, UK), beta‐actin (ab6276, Abcam, UK); mouse anti‐MCM2 (BM‐28, BD Biosciences, UK) and rabbit anti‐MCM2 (SP85) from Sigma; pSAMHD1 ProSci (Poway, CA, USA); p53 (GTX100629, GeneTex, Irvine, CA, USA); p‐p53 (9286P, Cell Signaling Technology); H2AX (613301, BioLegend, San Diego, CA, USA); γH2AX (613402, BioLegend); p21(sc‐6246, Santa Cruz Biotechnology); p27 (GTX100446, GeneTex); PARP (9542T, Cell Signaling Technology); IRF3 (11904P, Cell Signaling Technology); and 53BP1 (612522, BD Biosciences, UK). pCDNA Vpx Q76A was kind gift from J. Luban (Reinhard *et al*, 2014).

### Cell lines and viruses

293T cells were cultured in DMEM complete (DMEM supplemented with 100 U/ml penicillin, 0.1 mg/ml streptomycin and 10% FCS). SIVmac virus‐like particles containing Vpx were prepared as previously described (Goujon *et al*, 2008). VSV‐G HIV‐1 GFP virus was produced by transfection of 293T with GFP‐encoding genome CSGW, packaging plasmid p8.91 and pMDG as previously described (Besnier *et al*, 2002). Stocks of macrophage tropic virus BaL were prepared by infecting a single preparation of MDM. Cell‐free supernatants were collected from the infected cultures and stored in liquid nitrogen until further use. Virus stocks were titrated using the focus‐forming assay.

HSV‐1 KOS K26GFP encoding a VP26‐green fluorescent protein (GFP) fusion protein (Blondeau *et al*, 2013). Ad‐gfp, adenovirus with E1 and E3 deletion, insertion of CMV‐driven gfp in E1 region was produced by Native Antigen Company, Oxfordshire. SFV was kind gift from M. Mazzon (UCL).

### Focus‐forming assay

NP‐2 CD4/CCR5 cells were plated in 48‐well plates at a density of 1.5 × 10^4^ cells/well, 1 day before infection. Cells were infected for 4 h, washed and cultivated for 60 h in DMEM containing 10% FCS. Cells were fixed in methanol/acetone (1:1) and incubated for 1 h with a mixture of two anti‐HIV‐1 p24 mAbs (ARP365/366) diluted in PBS/1% FCS, washed in PBS/1% FCS and incubated for 1 h with a secondary anti‐mouse β‐galactosidase‐coupled antibody. After washing with PBS/1% FCS, β‐galactosidase substrate solution (0.5 mg/ml 5‐bromo‐4‐chloro‐3‐indolyl‐β‐galactoside in PBS containing 3 mM potassium ferricyanide and 1 mM magnesium chloride) was added, and the cells were incubated overnight. Blue‐stained infected cell foci were counted microscopically, and virus titres were expressed as ffu/ml.

### Monocyte isolation and differentiation

PBMC were prepared from HIV seronegative donors (after informed consent was obtained), by density‐gradient centrifugation (Lymphoprep, Axis‐Shield, UK). Monocyte‐derived macrophages (MDM) were prepared by adherence with washing of non‐adherent cells after 2 h, with subsequent maintenance of adherent cells in RPMI 1640 medium supplemented with 10% human serum and MCSF (10 ng/ml) for 3 days and then differentiated for a further 4 days in RPMI 1640 medium supplemented with 10% fetal calf sera without M‐CSF.

### Infection of primary cells using full‐length and VSV‐G pseudotyped HIV‐1 viruses

Macrophage tropic virus BaL was added to MDM and after 4‐h incubation removed and cells were washed in culture medium. Cells were fixed in 3% PFA, permeabilized by saponin and stained for intracellular p24 using anti‐p24 FITC‐conjugated antibody (Santa Cruz Biotechnology, USA). The percentage of infected cells was monitored by flow cytometry using BD FACSCalibur (BD Biosciences, UK) and analysed by CellQuest (BD Biosciences) and FlowJo software (Tree Star, OR, USA). GFP containing VSV‐G pseudotyped HIV‐1 was added to MDM and after 4‐h incubation removed and cells were washed in culture medium. The percentage of infected cells was determined 48 h post‐infection by flow cytometry using BD FACSCalibur (BD Biosciences, UK) and analysed by CellQuest (BD Biosciences) and FlowJo software (Tree Star, OR, USA) or by Hermes WiScan automated cell‐imaging system (IDEA Bio‐Medical Ltd. Rehovot, Israel) and analysed using MetaMorph and ImageJ software.

### Quantitative PCR for total HIV DNA quantitation

Total HIV DNA was detected as previously described (Mlcochova *et al*, 2014). Reverse transcription inhibitor AZT (20 μM) was used as a control to ensure that the total viral DNA measured was the product of productive infection and not a result of DNA contamination of the viral stocks.

### Integrated DNA quantitation

Integrated HIV DNA was measured as described (Liszewski *et al*, 2009). In total, 7,500 cells were assayed per well with five replicates and three independent experiments.

### qPCR for detection of 2‐LTR circles

2 × 10^5^ MDM were infected with 100 ng of p24 of DNaseI‐treated viruses. Cells were washed and harvested for DNA isolation 24 h post‐infection. 2‐LTR circles were quantified in triplicate by qPCR as described (Apolonia *et al*, 2007). Primers and probe used to detect 2‐LTR circles were as follows: 5′‐AACTAGAGATCCCTCAGACCCTTTT‐3′ and 5′‐CTTGTCTTCGTTGGGAGTGAATT‐3′, and TaqMan probe 5′‐FAM‐CTAGAGATTTTCCACACTGAC‐TAMRA‐3′.

### SDS–PAGE and immunoblots

Cells were lysed in reducing Laemmli SDS sample buffer containing PhosSTOP (Phosphatase Inhibitor Cocktail Tablets, Roche, Switzerland) at 96°C for 10 min and the proteins separated on NuPAGE^®^ Novex^®^ 4‐12% Bis–Tris gels. Subsequently, the proteins were transferred onto PVDF membranes (Millipore, Billerica, MA, USA), the membranes were quenched, and proteins were detected using specific antibodies. Labelled protein bands were detected using Amersham ECL Prime Western Blotting Detection Reagent (GE Healthcare, USA) and Amersham Hyperfilm or AlphaInnotech CCD camera. Protein band intensities were recorded and quantified using AlphaInnotech CCD camera and AlphaView software (Protein Simple, San Jose, California, USA).

### SAMHD1 knock‐down by siRNA

1 × 10^5^ MDM differentiated in MCSF for 4 days were transfected with 20 pmol of siRNA (L‐013950‐01, Dharmacon) using Lipofectamine RNAiMAX Transfection Reagent (Invitrogen). Transfection medium was replaced after 18 h with RPMI 1640 medium supplemented with 10% FCS and cells cultured for additional 3 days before infection.

### Quantitative PCR–TaqMan

Total RNA was purified from cell lysates collected in RLT buffer (Qiagen) using the RNEasy Mini kit (Qiagen). cDNA was synthesized using the Omniscript RT Kit (Qiagen), and quantitative (q)PCR of selected genes was performed using the follow ing inventoried TaqMan assays (Applied Biosystems);

CXCL10: forward 5′‐TGGCATTCAAGGAGTACCTC‐3′ and reverse 5′‐TTGTAGCAATGATCTCAACACG‐3′. MxA: forward 5′‐ATCCTGGGATTTTGGGGCTT‐3′ and reverse 5′‐CCGCTTGTCGCTGGTGTCG‐3′. ISG56: forward 5′‐CCTCCTTGGGTTCGTCTACA‐3′ and reverse 5′‐GGCTGATATCTGGGTGCCTA‐3′. ISG54: forward 5′‐CAGCTGAGAATTGCACTGCAA‐3′ and reverse 5′‐CGTAGGCTGCTCTCCAAGGA‐3′. IRF1: forward 5′‐TCTTAGCATCTCGGCTGGACTTC‐3′ and reverse 5′‐CGATACAAAGCAGGGGAAAAGG‐3′. IRF7: forward 5′‐GATGTCGTCATAGAGGCTGTTGG‐3′ and reverse 5′‐TGGTCC TGGTGAAGCTGGAA‐3′. STING: forward 5′‐TGGGGTGCCTGATAAC‐3′ and reverse 5′‐TGGCAAACAAAGTCTG‐3′. cGAS: forward 5′‐GGGAGCCCTGCTGTAACACTTCTTAT‐3′ and reverse 5′‐TTTGCATGCTTGGGTACAAGGT‐3′.

Expression levels of target genes were normalized to glyceraldehyde‐3‐phosphate dehydrogenase (GAPDH) as previously described (Tsang *et al*, 2009).

### Measurement of deoxynucleoside triphosphate levels in cells

dTTP and dCTP levels were measured essentially as previously described (Arnold *et al*, 2015). Briefly, deoxynucleoside triphosphates were extracted from batches of 1 × 10^6^ untreated and ETO‐treated macrophages according to del Val *et al* (2013). The dNTP levels were quantified by radiolabel incorporation assays performed using oligonucleotide templates detailed in Sherman & Fyfe (1989) and the procedures described in Ferraro *et al* (2010) with the following modifications. Standard curves ranged from 50 to 8,000 fmole, 5 units of Taq polymerase (Invitrogen) was used, and 2.5 μM of α‐^32^P‐dATP was employed as an incorporation label.

### Immunofluorescence

MDM were fixed in 3% PFA, quenched with 50 mM NH_4_Cl and permeabilized with 0.1% Triton X‐100 in PBS or 90% methanol. After blocking in PBS/1% FCS, MDM were labelled for 1 h with primary antibodies diluted in PBS/1% FCS, washed and labelled again with Alexa Fluor secondary antibodies for 1 h. Cells were washed in PBS/1% FCS and stained with DAPI in PBS for 20 min. Labelled cells were detected using Hermes WiScan automated cell‐imaging system (IDEA Bio‐Medical Ltd. Rehovot, Israel) and analysed using MetaMorph and ImageJ software.

### Ethics statement

Adult subjects provided written informed consent. Primary Macrophage & Dendritic Cell Cultures from Healthy Volunteer Blood Donors has been reviewed and granted ethical permission by the National Research Ethics Service through The Joint UCL/UCLH Committees on the Ethics of Human Research (Committee Alpha) 2nd of December 2009. Reference number 06/Q0502/92.

## Author contributions

PM, RKG and GJT designed experiments; PM, RKG and GJT wrote the manuscript; PM performed experiments; PM, RKG and GJT analysed data. SJC and IAT designed, performed experiments and analysed dNTP data.

## Conflict of interest

The authors declare that they have no conflict of interest.

## Supporting information



Expanded View Figures

Review Process File

Source Data for Figure 1

Source Data for Figure 2

Source Data for Figure 3

Source Data for Figure 5

## References

[embj201796880-bib-0001] Alexaki A , Liu YJ , Wigdahl B (2008) Cellular reservoirs of HIV‐1 and their role in viral persistence. Curr HIV Res 6: 388–400 18855649 10.2174/157016208785861195 PMC2683678

[embj201796880-bib-0002] Antonucci JM , St Gelais C , de Silva S , Yount JS , Tang C , Ji X , Shepard C , Xiong Y , Kim B , Wu L (2016) SAMHD1‐mediated HIV‐1 restriction in cells does not involve ribonuclease activity. Nat Med 22: 1072–1074 27711056 10.1038/nm.4163 PMC5069697

[embj201796880-bib-0003] Apolonia L , Waddington SN , Fernandes C , Ward NJ , Bouma G , Blundell MP , Thrasher AJ , Collins MK , Philpott NJ (2007) Stable gene transfer to muscle using non‐integrating lentiviral vectors. Mol Ther 15: 1947–1954 17700544 10.1038/sj.mt.6300281

[embj201796880-bib-0004] Ariumi Y , Turelli P , Masutani M , Trono D (2005) DNA damage sensors ATM, ATR, DNA‐PKcs, and PARP‐1 are dispensable for human immunodeficiency virus type 1 integration. J Virol 79: 2973–2978 15709017 10.1128/JVI.79.5.2973-2978.2005 PMC548471

[embj201796880-bib-0005] Arnold LH , Groom HC , Kunzelmann S , Schwefel D , Caswell SJ , Ordonez P , Mann MC , Rueschenbaum S , Goldstone DC , Pennell S , Howell SA , Stoye JP , Webb M , Taylor IA , Bishop KN (2015) Phospho‐dependent regulation of SAMHD1 oligomerisation couples catalysis and restriction. PLoS Pathog 11: e1005194 26431200 10.1371/journal.ppat.1005194 PMC4592219

[embj201796880-bib-0006] Badia R , Pujantell M , Riveira‐Munoz E , Puig T , Torres‐Torronteras J , Marti R , Clotet B , Ampudia RM , Vives‐Pi M , Este JA , Ballana E (2016) The G1/S specific cyclin D2 is a regulator of HIV‐1 restriction in non‐proliferating cells. PLoS Pathog 12: e1005829 27541004 10.1371/journal.ppat.1005829 PMC4991798

[embj201796880-bib-0007] Behrendt R , Schumann T , Gerbaulet A , Nguyen LA , Schubert N , Alexopoulou D , Berka U , Lienenklaus S , Peschke K , Gibbert K , Wittmann S , Lindemann D , Weiss S , Dahl A , Naumann R , Dittmer U , Kim B , Mueller W , Gramberg T , Roers A (2013) Mouse SAMHD1 has antiretroviral activity and suppresses a spontaneous cell‐intrinsic antiviral response. Cell Rep 4: 689–696 23972988 10.1016/j.celrep.2013.07.037 PMC4807655

[embj201796880-bib-0008] Besnier C , Takeuchi Y , Towers G (2002) Restriction of lentivirus in monkeys. Proc Natl Acad Sci USA 99: 11920–11925 12154231 10.1073/pnas.172384599 PMC129369

[embj201796880-bib-0009] Bhattacharya A , Wang Z , White T , Buffone C , Nguyen LA , Shepard CN , Kim B , Demeler B , Diaz‐Griffero F , Ivanov DN (2016) Effects of T592 phosphomimetic mutations on tetramer stability and dNTPase activity of SAMHD1 cannot explain the retroviral restriction defect. Sci Rep 6: 31353 27511536 10.1038/srep31353 PMC4980677

[embj201796880-bib-0010] Blondeau C , Pelchen‐Matthews A , Mlcochova P , Marsh M , Milne RSB , Towers GJ (2013) Tetherin restricts herpes simplex virus 1 and is antagonized by glycoprotein M. J Virol 87: 13124–13133 24067975 10.1128/JVI.02250-13 PMC3838283

[embj201796880-bib-0011] Branzei D , Foiani M (2008) Regulation of DNA repair throughout the cell cycle. Nat Rev Mol Cell Biol 9: 297–308 18285803 10.1038/nrm2351

[embj201796880-bib-0012] Brzostek‐Racine S , Gordon C , Van Scoy S , Reich NC (2011) The DNA damage response induces IFN. J Immunol 187: 5336–5345 22013119 10.4049/jimmunol.1100040 PMC3246365

[embj201796880-bib-0013] Clifford R , Louis T , Robbe P , Ackroyd S , Burns A , Timbs AT , Colopy GW , Dreau H , Sigaux F , Judde JG , Rotger M , Telenti A , Lin YL , Pasero P , Maelfait J , Titsias M , Cohen DR , Henderson SJ , Ross MT , Bentley D *et al* (2014) SAMHD1 is mutated recurrently in chronic lymphocytic leukemia and is involved in response to DNA damage. Blood 123: 1021–1031 24335234 10.1182/blood-2013-04-490847 PMC3924925

[embj201796880-bib-0014] Cribier A , Descours B , Valadao AL , Laguette N , Benkirane M (2013) Phosphorylation of SAMHD1 by cyclin A2/CDK1 regulates its restriction activity toward HIV‐1. Cell Rep 3: 1036–1043 23602554 10.1016/j.celrep.2013.03.017

[embj201796880-bib-0015] Crow YJ , Manel N (2015) Aicardi‐Goutieres syndrome and the type I interferonopathies. Nat Rev Immunol 15: 429–440 26052098 10.1038/nri3850

[embj201796880-bib-0016] Daniel R , Katz RA , Skalka AM (1999) A role for DNA‐PK in retroviral DNA integration. Science 284: 644–647 10213687 10.1126/science.284.5414.644

[embj201796880-bib-0017] Daniel R , Kao G , Taganov K , Greger JG , Favorova O , Merkel G , Yen TJ , Katz RA , Skalka AM (2003) Evidence that the retroviral DNA integration process triggers an ATR‐dependent DNA damage response. Proc Natl Acad Sci USA 100: 4778–4783 12679521 10.1073/pnas.0730887100 PMC153632

[embj201796880-bib-0018] Daniel R , Ramcharan J , Rogakou E , Taganov KD , Greger JG , Bonner W , Nussenzweig A , Katz RA , Skalka AM (2004) Histone H2AX is phosphorylated at sites of retroviral DNA integration but is dispensable for postintegration repair. J Biol Chem 279: 45810–45814 15308627 10.1074/jbc.M407886200

[embj201796880-bib-0019] De Iaco A , Santoni F , Vannier A , Guipponi M , Antonarakis S , Luban J (2013) TNPO3 protects HIV‐1 replication from CPSF6‐mediated capsid stabilization in the host cell cytoplasm. Retrovirology 10: 20 23414560 10.1186/1742-4690-10-20 PMC3599327

[embj201796880-bib-0020] DeHart JL , Andersen JL , Zimmerman ES , Ardon O , An DS , Blackett J , Kim B , Planelles V (2005) The ataxia telangiectasia‐mutated and Rad3‐related protein is dispensable for retroviral integration. J Virol 79: 1389–1396 15650165 10.1128/JVI.79.3.1389-1396.2005 PMC544104

[embj201796880-bib-0021] Ebina H , Kanemura Y , Suzuki Y , Urata K , Misawa N , Koyanagi Y (2012) Integrase‐independent HIV‐1 infection is augmented under conditions of DNA damage and produces a viral reservoir. Virology 427: 44–50 22374236 10.1016/j.virol.2012.02.004

[embj201796880-bib-0022] Ferraro P , Franzolin E , Pontarin G , Reichard P , Bianchi V (2010) Quantitation of cellular deoxynucleoside triphosphates. Nucleic Acids Res 38: e85 20008099 10.1093/nar/gkp1141 PMC2847218

[embj201796880-bib-0023] Goldstone DC , Ennis‐Adeniran V , Hedden JJ , Groom HCT , Rice GI , Christodoulou E , Walker PA , Kelly G , Haire LF , Yap MW , de Carvalho LPS , Stoye JP , Crow YJ , Taylor IA , Webb M (2011) HIV‐1 restriction factor SAMHD1 is a deoxynucleoside triphosphate triphosphohydrolase. Nature 480: 379–U134 22056990 10.1038/nature10623

[embj201796880-bib-0024] Gorczyca W , Bigman K , Mittelman A , Ahmed T , Gong J , Melamed MR , Darzynkiewicz Z (1993) Induction of DNA strand breaks associated with apoptosis during treatment of leukemias. Leukemia 7: 659–670 8483318

[embj201796880-bib-0025] Goujon C , Arfi V , Pertel T , Luban J , Lienard J , Rigal D , Darlix JL , Cimarelli A (2008) Characterization of simian immunodeficiency virus SIVSM/human immunodeficiency virus type 2 Vpx function in human myeloid cells. J Virol 82: 12335–12345 18829761 10.1128/JVI.01181-08 PMC2593360

[embj201796880-bib-0026] Hartlova A , Erttmann SF , Raffi FA , Schmalz AM , Resch U , Anugula S , Lienenklaus S , Nilsson LM , Kroger A , Nilsson JA , Ek T , Weiss S , Gekara NO (2015) DNA damage primes the type I interferon system via the cytosolic DNA sensor STING to promote anti‐microbial innate immunity. Immunity 42: 332–343 25692705 10.1016/j.immuni.2015.01.012

[embj201796880-bib-0027] Honeycutt JB , Wahl A , Baker C , Spagnuolo RA , Foster J , Zakharova O , Wietgrefe S , Caro‐Vegas C , Madden V , Sharpe G , Haase AT , Eron JJ , Garcia JV (2016) Macrophages sustain HIV replication *in vivo* independently of T cells. J Clin Invest 126: 1353–1366 26950420 10.1172/JCI84456 PMC4811134

[embj201796880-bib-0028] Hrecka K , Hao C , Gierszewska M , Swanson SK , Kesik‐Brodacka M , Srivastava S , Florens L , Washburn MP , Skowronski J (2011) Vpx relieves inhibition of HIV‐1 infection of macrophages mediated by the SAMHD1 protein. Nature 474: 658–661 21720370 10.1038/nature10195 PMC3179858

[embj201796880-bib-0029] Hsiang YH , Lihou MG , Liu LF (1989) Arrest of replication forks by drug‐stabilized topoisomerase I‐DNA cleavable complexes as a mechanism of cell killing by camptothecin. Can Res 49: 5077–5082 2548710

[embj201796880-bib-0030] Jackson SP , Bartek J (2009) The DNA‐damage response in human biology and disease. Nature 461: 1071–1078 19847258 10.1038/nature08467 PMC2906700

[embj201796880-bib-0031] Kim ET , White TE , Brandariz‐Nunez A , Diaz‐Griffero F , Weitzman MD (2013) SAMHD1 restricts herpes simplex virus 1 in macrophages by limiting DNA replication. J Virol 87: 12949–12956 24067963 10.1128/JVI.02291-13 PMC3838123

[embj201796880-bib-0032] Koyama T , Sun B , Tokunaga K , Tatsumi M , Ishizaka Y (2013) DNA damage enhances integration of HIV‐1 into macrophages by overcoming integrase inhibition. Retrovirology 10: 21 23432899 10.1186/1742-4690-10-21 PMC3605128

[embj201796880-bib-0033] Kretschmer S , Wolf C , Konig N , Staroske W , Guck J , Hausler M , Luksch H , Nguyen LA , Kim B , Alexopoulou D , Dahl A , Rapp A , Cardoso MC , Shevchenko A , Lee‐Kirsch MA (2015) SAMHD1 prevents autoimmunity by maintaining genome stability. Ann Rheum Dis 74: e17 24445253 10.1136/annrheumdis-2013-204845 PMC4345975

[embj201796880-bib-0034] Laguette N , Sobhian B , Casartelli N , Ringeard M , Chable‐Bessia C , Segeral E , Yatim A , Emiliani S , Schwartz O , Benkirane M (2011) SAMHD1 is the dendritic‐ and myeloid‐cell‐specific HIV‐1 restriction factor counteracted by Vpx. Nature 474: 654–U132 21613998 10.1038/nature10117 PMC3595993

[embj201796880-bib-0035] Lahouassa H , Daddacha W , Hofmann H , Ayinde D , Logue EC , Dragin L , Bloch N , Maudet C , Bertrand M , Gramberg T , Pancino G , Priet S , Canard B , Laguette N , Benkirane M , Transy C , Landau NR , Kim B , Margottin‐Goguet F (2012) SAMHD1 restricts the replication of human immunodeficiency virus type 1 by depleting the intracellular pool of deoxynucleoside triphosphates. Nat Immunol 13: 223–228 22327569 10.1038/ni.2236 PMC3771401

[embj201796880-bib-0036] Lau A , Swinbank KM , Ahmed PS , Taylor DL , Jackson SP , Smith GC , O'Connor MJ (2005) Suppression of HIV‐1 infection by a small molecule inhibitor of the ATM kinase. Nat Cell Biol 7: 493–500 15834407 10.1038/ncb1250

[embj201796880-bib-0037] Leonard B , Hart SN , Burns MB , Carpenter MA , Temiz NA , Rathore A , Vogel RI , Nikas JB , Law EK , Brown WL , Li Y , Zhang Y , Maurer MJ , Oberg AL , Cunningham JM , Shridhar V , Bell DA , April C , Bentley D , Bibikova M *et al* (2013) APOBEC3B upregulation and genomic mutation patterns in serous ovarian carcinoma. Cancer Res 73: 7222–7231 24154874 10.1158/0008-5472.CAN-13-1753 PMC3867573

[embj201796880-bib-0038] Li L , Olvera JM , Yoder KE , Mitchell RS , Butler SL , Lieber M , Martin SL , Bushman FD (2001) Role of the non‐homologous DNA end joining pathway in the early steps of retroviral infection. EMBO J 20: 3272–3281 11406603 10.1093/emboj/20.12.3272 PMC150207

[embj201796880-bib-0039] Liszewski MK , Yu JJ , O'Doherty U (2009) Detecting HIV‐1 integration by repetitive‐sampling Alu‐gag PCR. Methods 47: 254–260 19195495 10.1016/j.ymeth.2009.01.002 PMC2862469

[embj201796880-bib-0040] Little RF , Pittaluga S , Grant N , Steinberg SM , Kavlick MF , Mitsuya H , Franchini G , Gutierrez M , Raffeld M , Jaffe ES , Shearer G , Yarchoan R , Wilson WH (2003) Highly effective treatment of acquired immunodeficiency syndrome‐related lymphoma with dose‐adjusted EPOCH: impact of antiretroviral therapy suspension and tumor biology. Blood 101: 4653–4659 12609827 10.1182/blood-2002-11-3589

[embj201796880-bib-0041] Lusic M , Siliciano RF (2017) Nuclear landscape of HIV‐1 infection and integration. Nat Rev Microbiol 15: 69–82 27941817 10.1038/nrmicro.2016.162

[embj201796880-bib-0042] Mlcochova P , Watters SA , Towers GJ , Noursadeghi M , Gupta RK (2014) Vpx complementation of “non‐macrophage tropic” R5 viruses reveals robust entry of infectious HIV‐1 cores into macrophages. Retrovirology 11: 25 24656066 10.1186/1742-4690-11-25 PMC3997928

[embj201796880-bib-0043] Mlcochova P , Sutherland KA , Watters SA , Bertoli C , de Bruin RA , Rehwinkel J , Neil SJ , Lenzi GM , Kim B , Khwaja A , Gage MC , Georgiou C , Chittka A , Yona S , Noursadeghi M , Towers GJ , Gupta RK (2017) A G1‐like state allows HIV‐1 to bypass SAMHD1 restriction in macrophages. EMBO J 36: 604–616 28122869 10.15252/embj.201696025 PMC5331754

[embj201796880-bib-0044] Reinhard C , Bottinelli D , Kim B , Luban J (2014) Vpx rescue of HIV‐1 from the antiviral state in mature dendritic cells is independent of the intracellular deoxynucleotide concentration. Retrovirology 11: 12 24485168 10.1186/1742-4690-11-12 PMC3923257

[embj201796880-bib-0045] Roberts SA , Lawrence MS , Klimczak LJ , Grimm SA , Fargo D , Stojanov P , Kiezun A , Kryukov GV , Carter SL , Saksena G , Harris S , Shah RR , Resnick MA , Getz G , Gordenin DA (2013) An APOBEC cytidine deaminase mutagenesis pattern is widespread in human cancers. Nat Genet 45: 970–976 23852170 10.1038/ng.2702 PMC3789062

[embj201796880-bib-0046] Rothkamm K , Barnard S , Moquet J , Ellender M , Rana Z , Burdak‐Rothkamm S (2015) DNA damage foci: meaning and significance. Environ Mol Mutagen 56: 491–504 25773265 10.1002/em.21944

[embj201796880-bib-0047] Schmidt S , Schenkova K , Adam T , Erikson E , Lehmann‐Koch J , Sertel S , Verhasselt B , Fackler OT , Lasitschka F , Keppler OT (2015) SAMHD1's protein expression profile in humans. J Leukoc Biol 98: 5–14 25646359 10.1189/jlb.4HI0714-338RR PMC7166976

[embj201796880-bib-0048] Sherman PA , Fyfe JA (1989) Enzymatic assay for deoxyribonucleoside triphosphates using synthetic oligonucleotides as template primers. Anal Biochem 180: 222–226 2554751 10.1016/0003-2697(89)90420-x

[embj201796880-bib-0049] Siliciano RF , Greene WC (2011) HIV latency. Cold Spring Harb Perspect Med 1: a007096 22229121 10.1101/cshperspect.a007096 PMC3234450

[embj201796880-bib-0050] Skalka AM , Katz RA (2005) Retroviral DNA integration and the DNA damage response. Cell Death Differ 12(Suppl 1): 971–978 15761474 10.1038/sj.cdd.4401573

[embj201796880-bib-0051] Srivastava S , Swanson SK , Manel N , Florens L , Washburn MP , Skowronski J (2008) Lentiviral Vpx accessory factor targets VprBP/DCAF1 substrate adaptor for cullin 4 E3 ubiquitin ligase to enable macrophage infection. PLoS Pathog 4: e1000059 18464893 10.1371/journal.ppat.1000059 PMC2330158

[embj201796880-bib-0052] Sun LJ , Wu JX , Du FH , Chen X , Chen ZJJ (2013) Cyclic GMP‐AMP synthase is a cytosolic DNA sensor that activates the type I interferon pathway. Science 339: 786–791 23258413 10.1126/science.1232458 PMC3863629

[embj201796880-bib-0053] Tsang J , Chain BM , Miller RF , Webb BLJ , Barclay W , Towers GJ , Katz DR , Noursadeghi M (2009) HIV‐1 infection of macrophages is dependent on evasion of innate immune cellular activation. Aids 23: 2255–2263 19741482 10.1097/QAD.0b013e328331a4ce PMC2873676

[embj201796880-bib-0054] del Val IJ , Kyriakopoulos S , Polizzi KM , Kontoravdi C (2013) An optimized method for extraction and quantification of nucleotides and nucleotide sugars from mammalian cells. Anal Biochem 443: 172–180 24036437 10.1016/j.ab.2013.09.005

[embj201796880-bib-0055] Van Cor‐Hosmer SK , Kim DH , Daly MB , Daddacha W , Kim B (2013) Restricted 5′‐end gap repair of HIV‐1 integration due to limited cellular dNTP concentrations in human primary macrophages. J Biol Chem 288: 33253–33262 24097986 10.1074/jbc.M113.486787 PMC3829171

[embj201796880-bib-0056] Watters SA , Mlcochova P , Gupta RK (2013) Macrophages: the neglected barrier to eradication. Curr Opin Infect Dis 26: 561–566 24152764 10.1097/QCO.0000000000000014

[embj201796880-bib-0057] Welbourn SSK (2016) Low dNTP levels are necessary but may not be sufficient for lentiviral restriction by SAMHD1. Virology 15: 271–277 10.1016/j.virol.2015.11.022 PMC4744553 26655245

[embj201796880-bib-0058] White TE , Brandariz‐Nunez A , Valle‐Casuso JC , Amie S , Nguyen LA , Kim B , Tuzova M , Diaz‐Griffero F (2013) The retroviral restriction ability of SAMHD1, but not its deoxynucleotide triphosphohydrolase activity, is regulated by phosphorylation. Cell Host Microbe 13: 441–451 23601106 10.1016/j.chom.2013.03.005 PMC3864637

[embj201796880-bib-0059] Wu JX , Sun LJ , Chen X , Du FH , Shi HP , Chen C , Chen ZJJ (2013) Cyclic GMP‐AMP is an endogenous second messenger in innate immune signaling by cytosolic DNA. Science 339: 826–830 23258412 10.1126/science.1229963 PMC3855410

[embj201796880-bib-0060] Yang YX , Guen V , Richard J , Cohen EA , Berthoux L (2010) Cell context‐dependent involvement of ATR in early stages of retroviral replication. Virology 396: 272–279 19913868 10.1016/j.virol.2009.10.032 PMC3955184

