# Supplementary material for: DNA damage induced by topoisomerase inhibitors activates SAMHD1 and blocks HIV‐1 infection of macrophages
Source: EMBO J. 2017 Oct 30;37(1):50–62. doi: 10.15252/embj.201796880 (PMC5753034; doi:10.15252/embj.201796880)
Supplement: Supplementary file 1 — Expanded View Figures [file EMBJ-37-50-s001.pdf]

Expanded View Figures

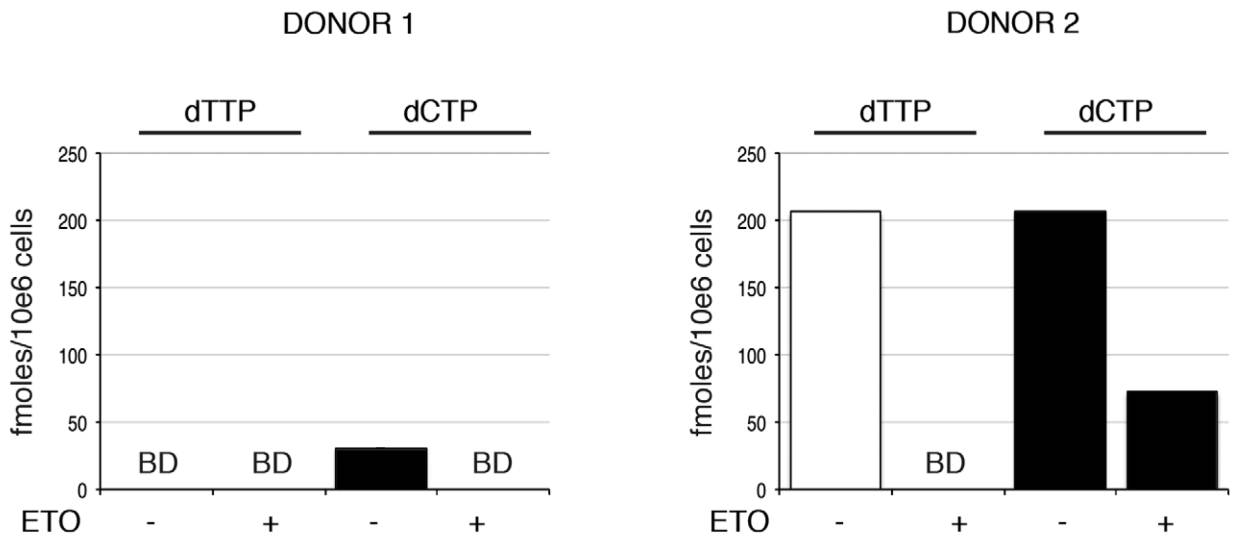

Figure EV1. Quantification of dNTP levels in MDM treated with etoposide (ETO).

Analysis of cellular dNTP levels in unstimulated and ETO-treated MDM. dNTPs were extracted for quantification from two donors. The bar graph shows the amounts (fmoles per 10^6 cells) of the indicated dNTP from each donor, with and without ETO treatment. BD: below level of detection.

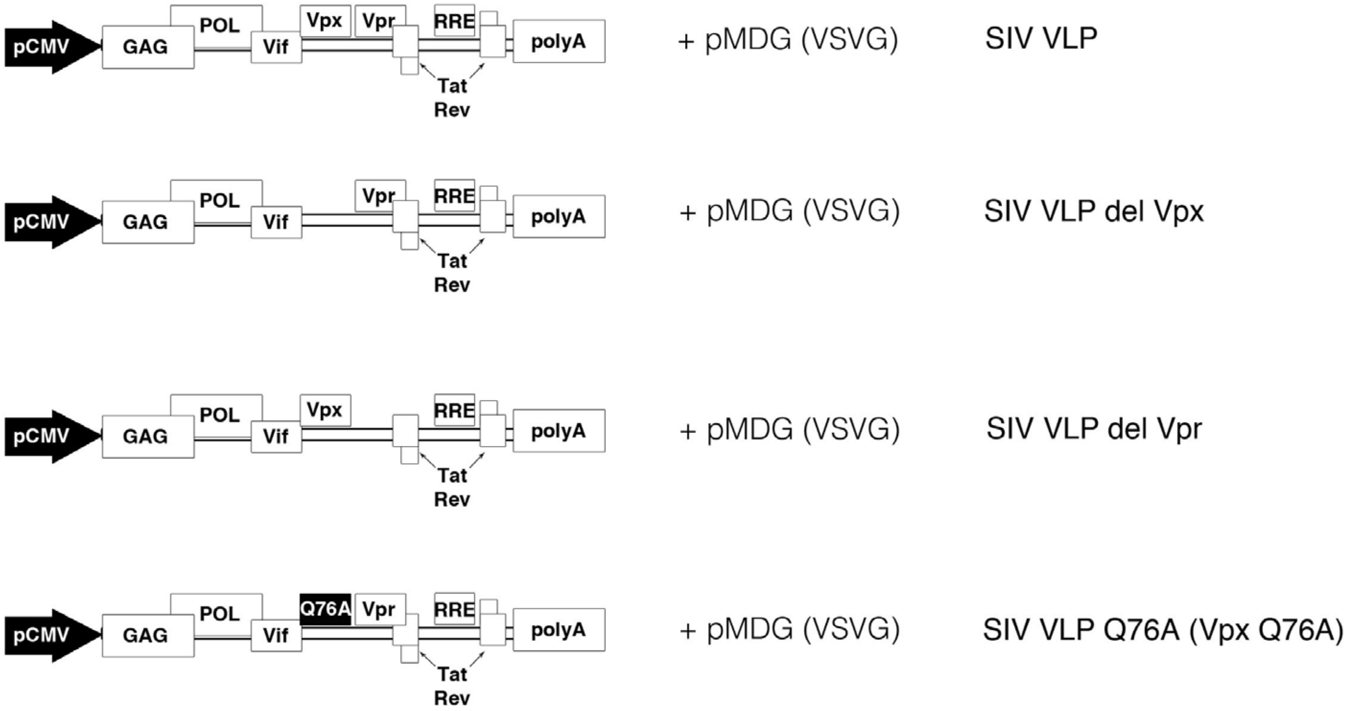

Figure EV2. Schematic representation of SIVmac VLP used in the study.

Schematic representation of the packaging plasmids derived from SIVmac251. SIVmac virus-like (SIV VLP) particles were prepared as described in Materials and Methods.

© 2017 The Authors

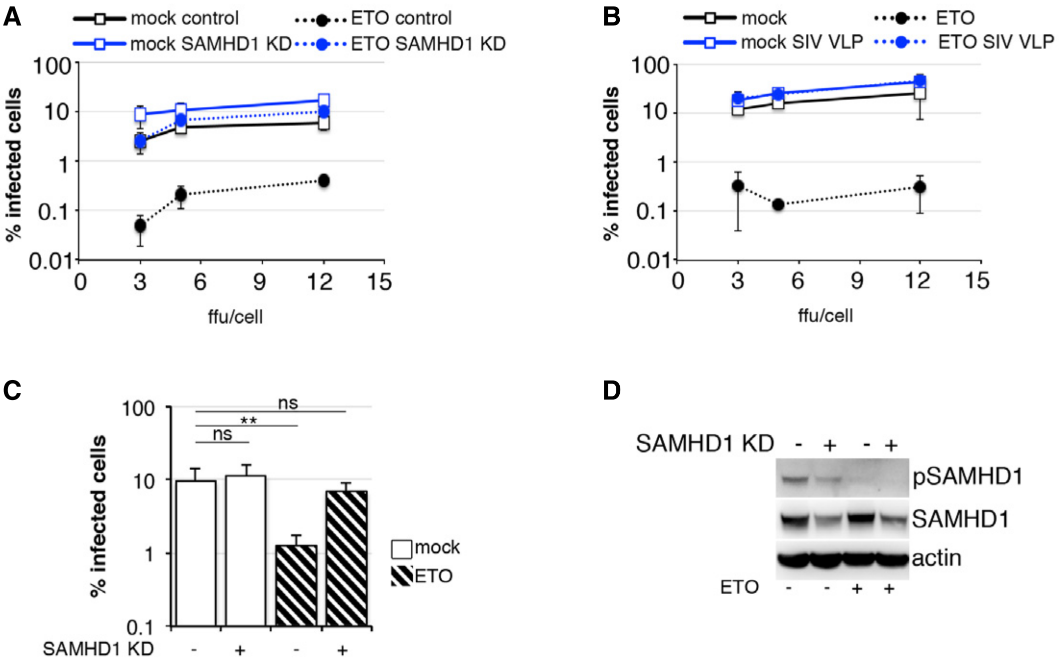

Figure EV3. SAMHD1 inhibits HIV-1 following DNA damage.

A MDM were transfected with control or pool of SAMHD1 (KD) siRNA and 3 days later treated with 5 μM ETO and infected in the presence of ETO with HIV-1 BaL (3, 5, 12 ffu/cell). Cells were stained for intracellular p24, and the percentage of infected cells was quantified 48 h post-infection by FACS (n = 3, mean ± s.e.m.).

B MDM were treated with 5 μM ETO for 18 h and co-infected in the presence of ETO with HIV-1 BaL (3, 5, 12 ffu/cell) and SIVmac virus-like particles containing Vpx/Vpr (SIV VLP). Cells were stained for intracellular p24, and the percentage of infected cells was quantified 48 h post-infection by FACS (n = 3, mean ± s.e.m.).

C MDM were transfected with control or pool of SAMHD1 (KD) siRNA and 3 days later treated with 5 μM ETO for 18 h and infected in the presence of ETO with VSV-G HIV-1 GFP. The percentage of infected cells was quantified by the automated cell-imaging system Hermes WiScan and ImageJ 48 h post-infection (n = 3, mean ± s.e.m.; **P-value ≤ 0.01; (ns) non-significant, paired t-test).

D Cells from a representative donor were used for immunoblotting.

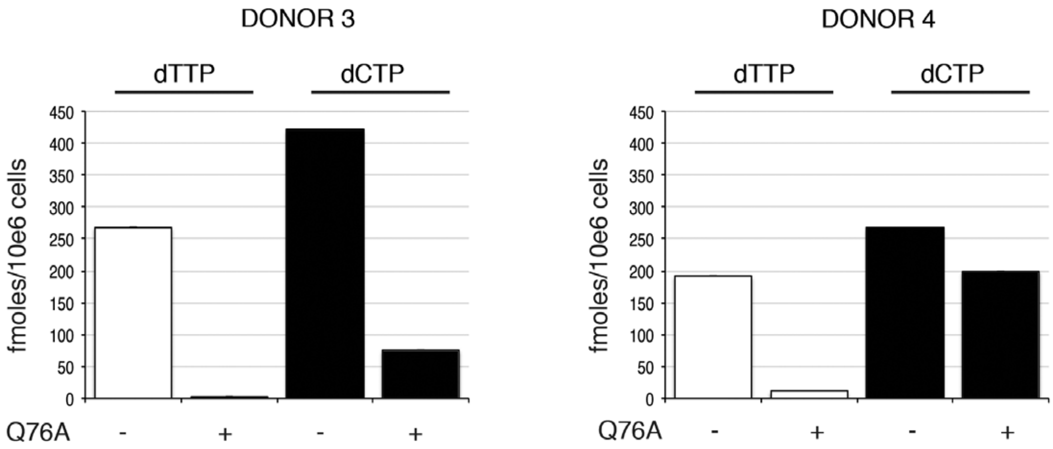

Figure EV4. Quantification of dNTP levels in MDM following treatment with SIV VLP Q76A.

Analysis of cellular dNTP levels in MDM and MDM expressing SIV Vpx(Q76A). dNTPs were extracted for quantification from two donors. The bar graph shows the amounts (fmoles per 10^6 cells) of the indicated dNTP from the MDM of each donor, before and after the introduction of SIV Vpx(Q76A) in SIV VLPs.

© 2017 The Authors
