# Supplementary figures and images for: DNA damage induced by topoisomerase inhibitors activates SAMHD1 and blocks HIV‐1 infection of macrophages
Source: EMBO J. 2017 Oct 30;37(1):50–62. doi: 10.15252/embj.201796880 (PMC5753034; doi:10.15252/embj.201796880)

Figure 1D

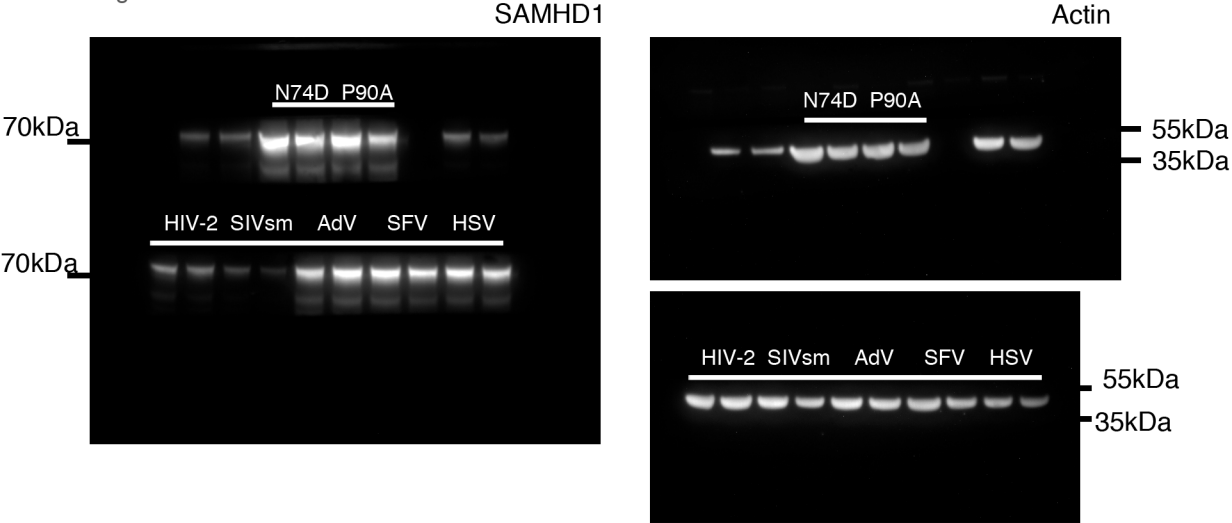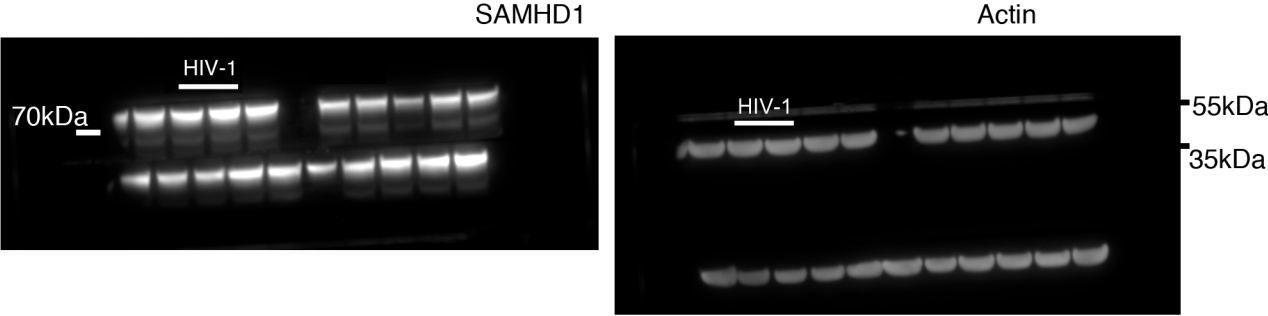

Supplement: Supplementary file 3 — Source Data for Figure 1 [file EMBJ-37-50-s002.pdf]

Figure 2A

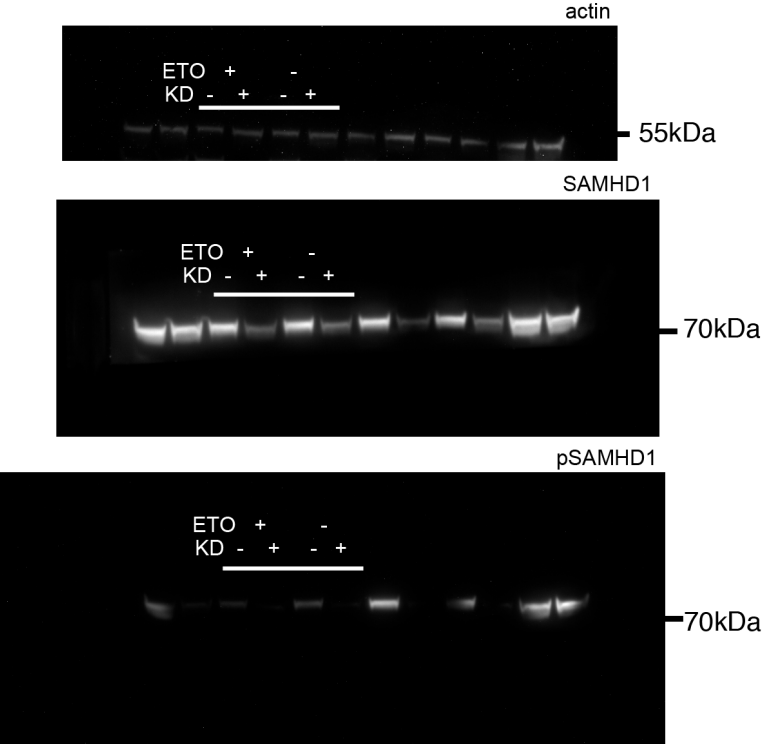

Figure 2B

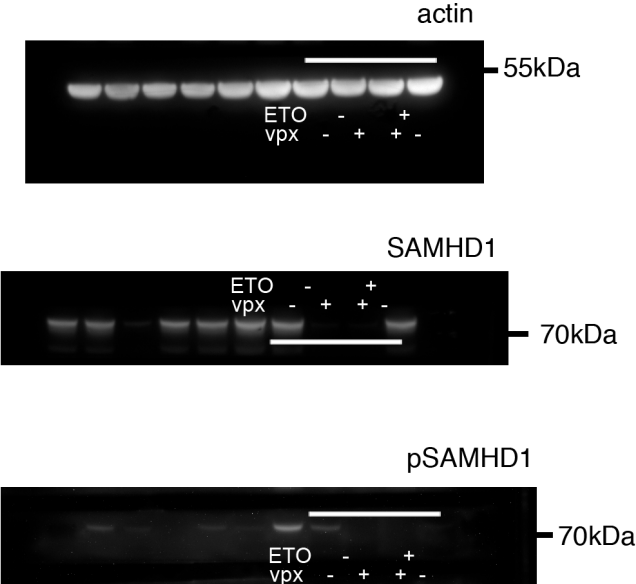

Figure 2C

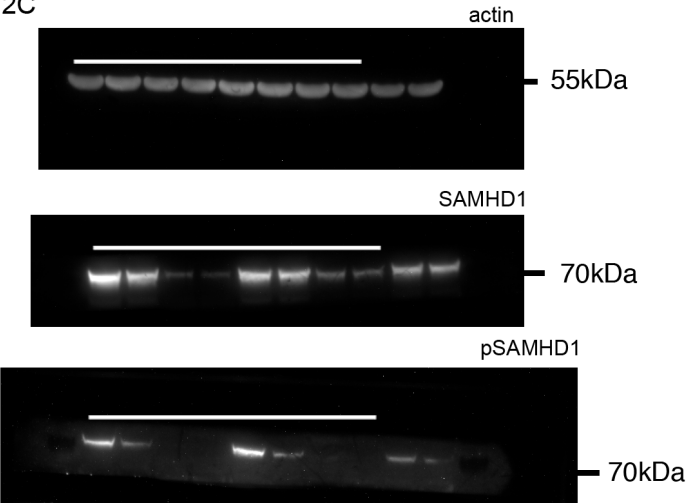

Supplement: Supplementary file 4 — Source Data for Figure 2 [file EMBJ-37-50-s003.pdf]

Figure 3C

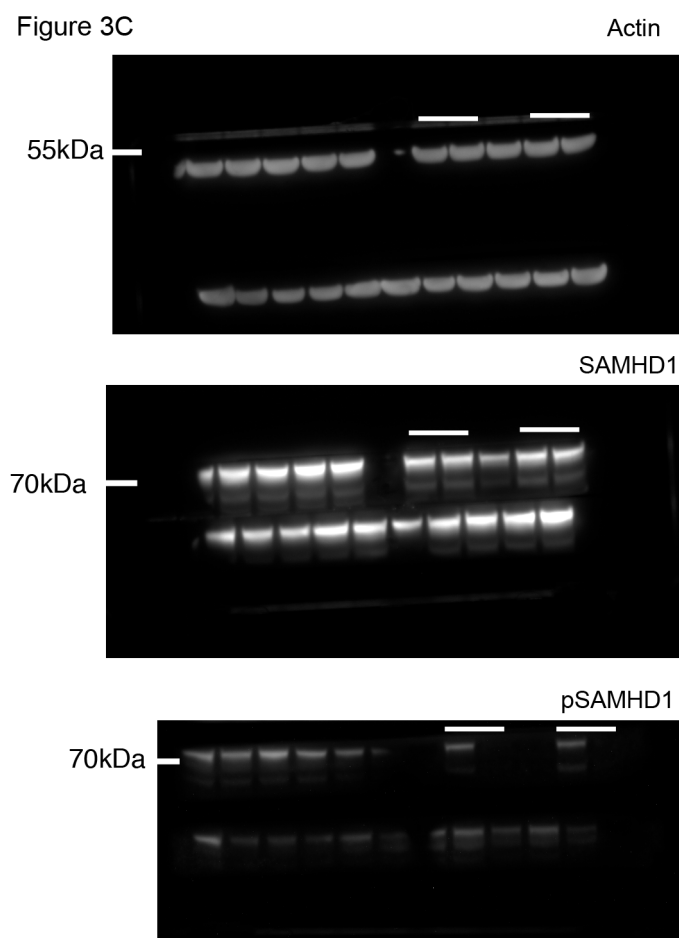

Supplement: Supplementary file 5 — Source Data for Figure 3 [file EMBJ-37-50-s004.pdf]

Figure 5B

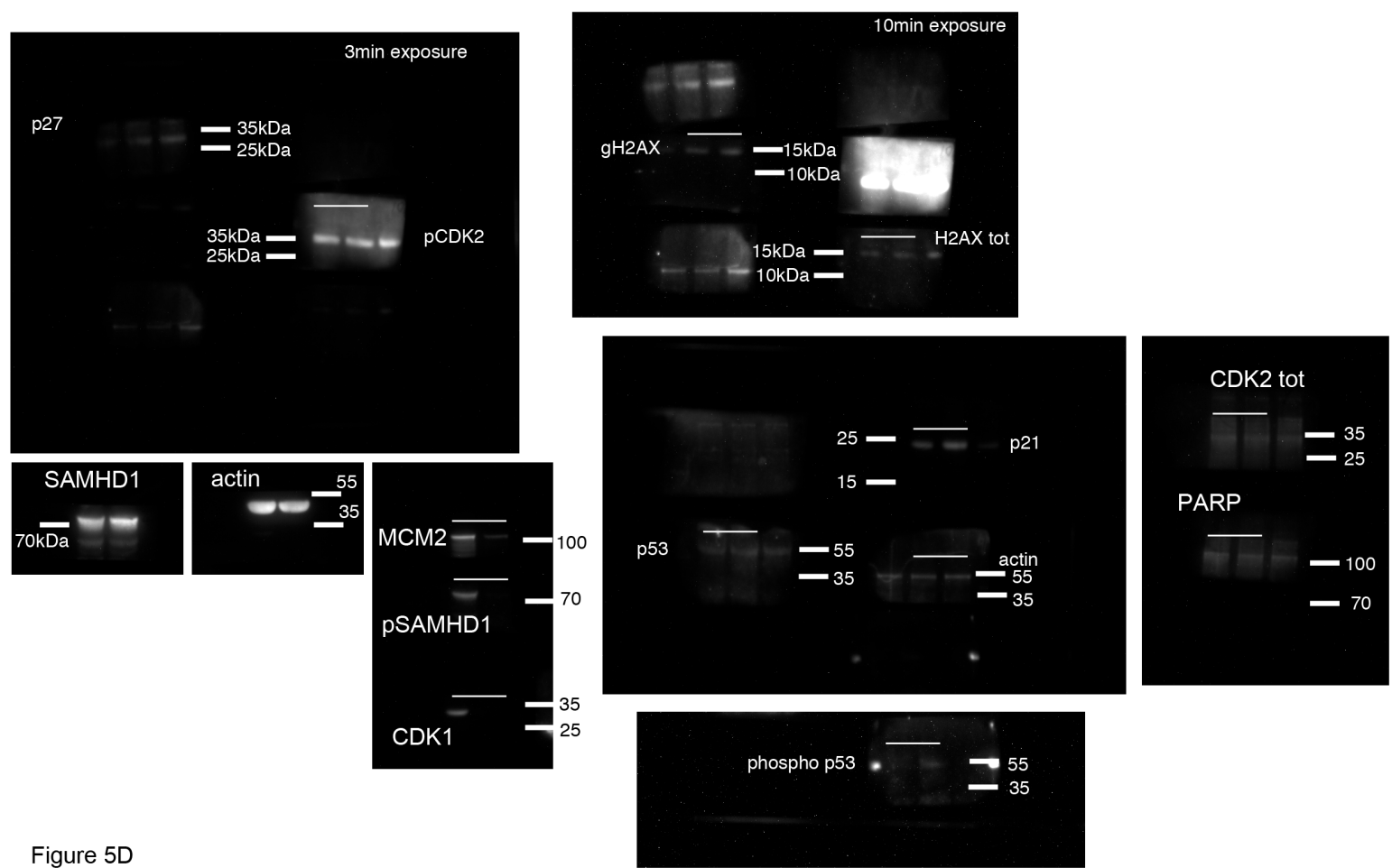

Figure 5D

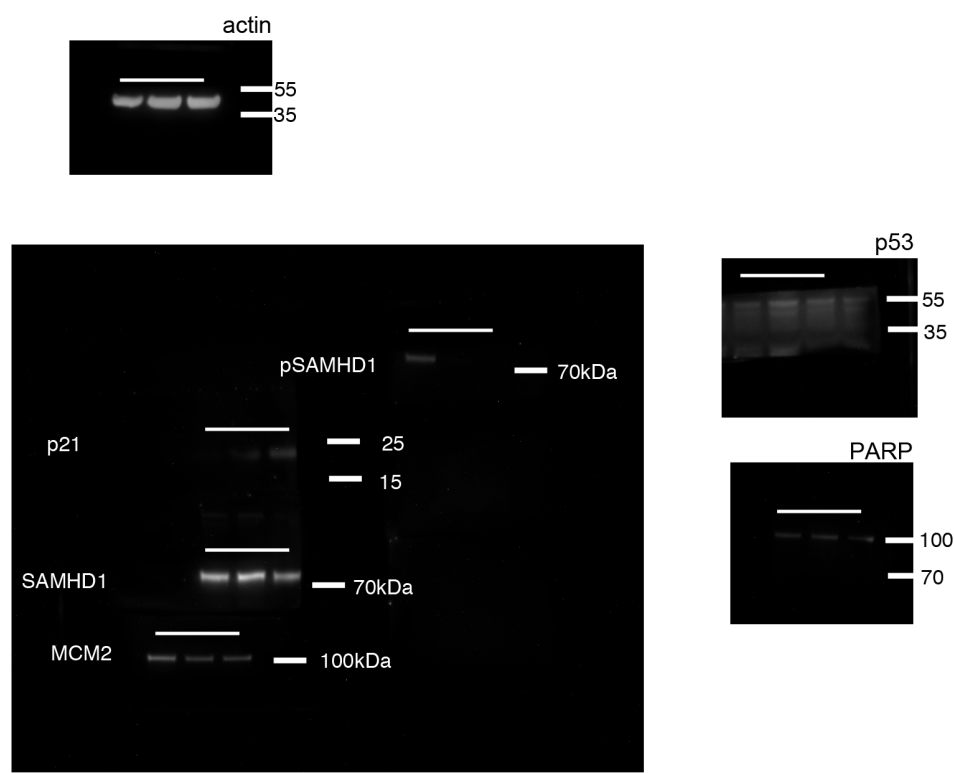

Supplement: Supplementary file 6 — Source Data for Figure 5 [file EMBJ-37-50-s005.pdf]
